# Rubimaillin ameliorates liver fibrosis by triggering the ferroptosis of activated hepatic stellate cells through targeting CPT1A

**DOI:** 10.7150/ijbs.120415

**Published:** 2026-01-22

**Authors:** Dingqi Zhang, Qingxuan Tang, Xiaoli He, Chengming Wen, Fengfeng Zhou, Xia Wei, Zikang Wang, Jiao Wang, Wei Liu, Ying Xu, Yunyao Jiang, Hang Yin

**Affiliations:** 1 State Key Laboratory of Membrane Biology, School of Pharmaceutical Sciences, Tsinghua-Peking Center for Life Sciences, Key Laboratory of Bioorganic Phosphorous Chemistry and Chemical Biology (Ministry of Education), Tsinghua University, 30 Shuangqing Road, Beijing 100084, China.; 2 Department of pharmacy, The NATCM Third Grade Laboratory of Traditional Chinese Medicine Preparations, Key Laboratory of Liver and Kidney Diseases (Ministry of Education), Shuguang Hospital Affiliated to Shanghai University of Traditional Chinese Medicine, 528 Zhangheng Road, Shanghai 201203, China.; 3 School of Traditional Chinese Medicine, Shanghai University of Traditional Chinese Medicine, 1200 Cailun Road, Shanghai 201203, China.; 4 Department of Endocrinology, Yueyang Hospital of Integrated Traditional Chinese and Western Medicine Affiliated to Shanghai University of Traditional Chinese Medicine, 110 Ganhe Road, Shanghai 200437, China.

**Keywords:** rubimaillin, liver fibrosis, hepatic stellate cells (HSCs), ferroptosis, carnitine palmitoyltransferase 1A (CPT1A)

## Abstract

Liver fibrosis is defined as the excessive accumulation of extracellular matrix proteins in the liver due to chronic liver injury. Targeted ferroptosis of activated hepatic stellate cells (HSCs) is considered a promising therapeutic strategy for liver fibrosis. Rubimaillin (Rub), a naphthoquinone compound extracted from traditional Chinese medicine *Rubia cordifolia L.*, exhibits various activities in multiple diseases. This study aimed to investigate the anti-hepatic fibrosis effect, the direct protein target, and molecular mechanism of Rub. Here, our results demonstrated that Rub effectively ameliorated liver fibrosis *via* triggering the ferroptosis of activated HSCs in mice models. Subsequently, we confirmed that Rub directly binds to carnitine palmitoyltransferase 1A (CPT1A) at SER592, THR594, and THR689, and inhibits its activity using PROTAC technology, computer molecular dynamics simulations, CETSA, DARTS, BLI, and site mutation assays. Further, the inhibition or deficiency of CPT1A in activated HSCs could trigger metabolic reprogramming-mediated ferroptosis. Moreover, CPT1A deficiency or overexpression could eliminate the effects of Rub-induced ferroptosis. Mechanistically, Rub-induced ferroptosis in activated HSCs was associated with metabolic reprogramming mediated by targeting CPT1A. Taken together, our results indicate the beneficial effects, the direct protein target and the molecular mechanism *via* which Rub induces ferroptosis in activated HSCs to ameliorate liver fibrosis.

## Introduction

Liver fibrosis is a crucial common pathological process in the progression of many chronic liver diseases. When the liver is continuously injured, such as being stimulated by pathogenic factors like chronic viral hepatitis (hepatitis B and hepatitis C), alcoholic liver disease, metabolic dysfunction-associated fatty liver disease (MAFLD), autoimmune liver disease, etc., hepatic stellate cells (HSCs) are activated, the synthesis of extracellular matrix (ECM) components increases while degradation decreases 1,2. Subsequently, a large amount of ECM is abnormally deposited in the liver tissue, gradually destroying the normal structure of the liver and making the liver texture become hard, thus forming liver fibrosis 3. With lifestyle changes, the incidence of alcoholic liver disease and MAFLD has risen sharply, coupled with the continued impact of chronic viral hepatitis, the prevalence of liver fibrosis has increased year by year 4. However, there is still an unmet clinical need in the field of liver fibrosis, and no specific chemical drugs and biological agents have been approved for its treatment 5,6. At present, the main approach is etiological treatment, for example, antiviral therapy in patients with chronic viral hepatitis, abstinence from alcohol in patients with alcoholic liver disease, weight control and improvement of metabolic disorders in patients with MAFLD 7-9. Given the limitations of existing treatments and the increasingly serious situation of liver fibrosis, there is an urgent need to find new therapeutic targets and drug candidates.

The occurrence of liver fibrosis is not caused by a single factor, but rather the result of the interweaving and synergistic effects of multiple cell types, signaling pathways, and extracellular environments 3. It is widely recognized that HSCs are the primary effector cells and their activation is recognized as a core event in the pathogenesis of liver fibrosis 10. In the normal liver, HSCs are in a quiescent state and their main function is to store vitamin A and maintain the normal structure of the liver. However, when the liver suffers sustained damage, various stimulation signals can disrupt the homeostasis of HSCs, prompting them to transform into myofibroblast-like cells 11. The activated HSCs have acquired robust proliferative and migratory capacities, contraction characteristics, and the ability to secrete cytokines and ECM 11,12. Notably, the proliferation and migration of HSCs require substantial energy input, thus triggering a cellular metabolic reprogramming 13. Carnitine palmitoyltransferase 1A (CPT1A), an enzyme located on the outer membrane of mitochondria, constitutes the rate limiting step of fatty acid oxidation by transporting long-chain fatty acids to mitochondria for β-oxidation, which generates energy in the form of ATP that plays a crucial role in regulating cellular energy metabolism 14. In addition, CPT1A mediated fatty acid oxidation to promotes cell proliferation by a nucleoside metabolism in nasopharyngeal carcinoma 15. It has been confirmed that the expression of CPT1A was significantly increased in HSCs in the mouse models of liver fibrosis and patients with liver fibrosis, and increased CPT1A could induce the activation of HSCs, thereby contributing to the progression of liver fibrosis 16. Conversely, pharmacological and genetic inhibition of CPT1A in HSCs could alleviate liver fibrosis by inhibiting the activation of HSCs 16,17. Therefore, the development of CPT1A-targeting compounds may open up new avenues for treating liver fibrosis.

As a new type of programmed cell death, ferroptosis has attracted much attention in the field of liver fibrosis study in recent years. Different from traditional apoptosis and necrosis, ferroptosis mainly depends on iron overload and lipid peroxidation accumulation, which then destroys the cell membrane structure and triggers cell death 18. Multiple studies have shown that in the process of liver fibrosis, the imbalance of iron metabolism in cells, the increase of iron ion uptake and the decrease of storage and output, lead to iron overload in HSCs and provide a hotbed for the occurrence of ferroptosis 19-21. Studies have confirmed that inducing activated HSCs ferroptosis could significantly decrease HSCs growth, inhibit ECM accumulation, and thereby alleviate liver fibrosis 22-24. Thus, inducing ferroptosis of activated HSCs is becoming a new and promising therapeutic strategy for liver fibrosis. Currently, CPT1A is an essential driver for ferroptosis resistance, blocking CPT1A can trigger ferroptosis 25-27. A recent study revealed that targeting CPT1A-mediated metabolic reprogramming to induce ferroptosis *via* two key mechanisms: down-regulating the nuclear factor erythroid 2-related factor 2 (NRF2)/Glutathione peroxidase 4 (GPX4) antioxidant system and enhancing long-chain acyl-coenzyme A synthase 4 (ACSL4)-mediated phospholipid polyunsaturated fatty acids (PL-PUFAs) production, both of which are regulated by c-Myc ubiquitination 25. Therefore, considering the role of CPT1A in activated HSCs and in ferroptosis, we speculate that CPT1A can also mediate metabolic remodeling to induce ferroptosis in activated HSCs, and thereby alleviating liver fibrosis. In brief, targeting CPT1A-mediated the ferroptosis of activated HSCs holds potential as a therapeutic strategy for liver fibrosis.

Currently, a variety of target fishing techniques are available for the components of traditional Chinese medicine (TCM) and natural products. Among these methods, affinity-based target identification captures targets through the inherent affinity between small molecules and proteins, which is characterized by simple operation yet is susceptible to non-specific binding events; activity-based protein profiling (ABPP) achieves specific binding to the active sites of proteins *via* activity probes, rendering it suitable for enzyme targets but limiting its application scope; Label-free target identification can circumvent label interference and recapitulate the physiological binding state of targets, yet it is hampered by inherent drawbacks including low sensitivity and high detection costs 28. In contrast to these conventional target-fishing technologies that primarily focus on “target recognition and identification”, PROTAC target-fishing technology—an advanced target-deconvolution tool—overcomes the limitations of traditional methods by avoiding chemical modification of natural products and enabling in situ target capture in live cells under physiological conditions. Consequently, this technology has been increasingly applied to the target identification of TCM components and natural products, offering a novel and efficient strategy for this research field 29-33. As a powerful endogenous protein degradation tool developed in recent years, PROTAC exerts its core advantage by not only specifically binding to target proteins but also triggering their ubiquitination and subsequent degradation *via* the ubiquitin-proteasome system 34, which further enhances the reliability of target identification.

*Rubia cordifolia L.* (roots or rhizomes) is a TCM that has been widely used in the treatment of arthritis, uterine hemorrhage, and chronic liver diseases in China. Rubimaillin (Rub, also known as Mollugin) is a naphthoquinone compound extracted from *Rubia cordifolia L*., which has exhibited various pharmacological activities in studies, such as anti-tumor, anti-inflammatory, and neuroprotective activities 35-37. In addition, it has been reported that Rub inhibits proliferation and drives ferroptosis in colorectal cancer cells 38. However, the role of Rub in liver fibrosis remains largely unclear. Given that CPT1A plays a central role in the activation of HSCs and their resistance to ferroptosis, and considering the potential of Rub in regulating ferroptosis, this study hypothesizes that Rub may exert an anti-liver fibrosis effect by targeting CPT1A in HSCs and thereby inducing their ferroptosis. In this study, to assess the anti-liver fibrosis of Rub, we established transforming growth factor-β1 (TGF-β1)-induced HSCs activation and carbon tetrachloride (CCl_4_)-induced liver fibrosis models. RNA sequencing (RNA-seq) analysis was performed to explore the potential mechanism, followed by validation experiments confirming Rub-induced ferroptosis in activated HSCs. PROTAC, docking, and binding assays identified and verified Rub interaction with CPT1A. CPT1A knockdown and overexpression models validated CPT1A-dependent ferroptosis by Rub. Finally, we analyzed Rub regulation of the NRF2/GPX4 and ACSL4/PL-PUFAs pathways to clarify its ferroptosis-inducing mechanism *via* CPT1A targeting.

## Materials and Methods

### Animals

Male C57BL/6J mice (6-8 weeks old) were purchased from Beijing Vital River Laboratory Animal Technology Co., Ltd. (Beijing, China). All experimental animals were housed and maintained under individually ventilated cage system (IVC) conditions with constant temperature and humidity and a 12-hour light/dark cycle. CCl_4_ model mice were fed and the relevant experiments were conducted in the Laboratory Animal Resources Center of Tsinghua University. This study protocol was approved and monitored by the Animal Ethics Committee of Tsinghua University (Ethics approval number: THU-02-2023-0029A).

### CCl_4_-induced liver fibrosis model

After one week of adaptive feeding, liver fibrosis model mice (*n* = 32) were established by intraperitoneal injection of 15% CCl_4_-olive oil solution at a dose of 2 ml/kg body weight thrice weekly for 6 weeks, and the final dose of CCl_4_ administered is 478.5 mg/kg body weight per injection 39. The control group mice (*n* = 8) were injected intraperitoneally with 2 ml/kg of olive oil. After the third week of CCl_4_ injection, CCl_4_-induced mice were randomly divided into four groups (*n* = 8): CCl_4_ group, Rub low-dose group (Rub-L, 10 mg/kg), Rub high-dose group (Rub-H, 20 mg/kg), and sorafenib group (Sora, 10 mg/kg). Mice were given corresponding doses of Rub and Sora treatment once a day, Rub and Sora were dissolved in 0.4% carboxymethyl cellulose sodium (CMC-Na) for oral administration. The control group and CCl_4_ group mice were given 0.4% CMC-Na. At the end of the 6th week, all mice were anesthetized, their liver tissues and serum samples were collected for subsequent experiments.

### Serum biochemistry, histological analysis and immunohistochemistry assays

Serum was obtained from blood samples through centrifugation at 3500 rpm for 15 min, and the serum levels of ALT and AST were detected using automatic biochemical analyzer at the Clinical Laboratory of Shuguang Hospital Affiliated to Shanghai University of Traditional Chinese Medicine (Shanghai, China). The paraffin sections of liver tissue were sliced into 4 µm, H&E staining, Masson staining, Sirius red staining and immunohistochemical α-SMA staining were operated by Wuhan Servicebio Technology Co., Ltd. (Wuhan, China). The sections were scanned and photographed; additionally, the positive areas of Masson, Sirius red, and α-SMA immunohistochemical staining were further quantified by Leica LAS Image Analysis.

### Hepatic Hyp content assay

Hepatic Hyp content was detected using alkaline water method according to the manufacturers'instructions of the reagent kit. Briefly, liver tissues (50 mg) were weighed, hydrolyzed, and adjusted for pH value (6.0-6.8), as well as the solution was added with corresponding reagent. Subsequently, the samples were tested with a microscope (BioTek, Hercules, CA, USA).

### Cell culture and treatment

HSCs line LX2 and JS1 cells were cultured in DMEM supplemented with 10% FBS and 1% double antibody. Hepatocyte line AML12 cells were cultured in DMEM-F12 supplemented with 10% FBS and 1% double antibody. Human hepatic sinusoidal endothelial cells (HHSEC) were cultured in the supplied complete ECM medium supplemented with basal medium, 5% FBS, 1% ECGS and 1% double antibody. Immortalized bone marrow-derived macrophages (iBMDM) were cultured in DMEM supplemented with 10% FBS and 1% double antibody. (i) LX2 and JS1 cells were seeded in 12-well plate, and were activated with transforming growth factor-β1 (TGF-β1, 10 ng/mL) and simultaneously treated with different concentrations of Rub (50, 100 and 150 μM) for 12 h, and then were collected by Trizol reagent for detection by qPCR assay. (ii) LX2 and JS1 cells were seeded in 6-well plates, and were activated with TGF-β1 (10 ng/mL) and were simultaneously treated with different concentrations of Rub (50, 100 and 150 μM) for 48h, and then were collected by RIPA solution for detection by western blot assay. (iii) LX2 cells were seeded in 6-well plates, and were incubated with Rub-PROTAC (20 μM) or DMSO or MG132 (10 μM) for 10 h, and then were collected for proteomic study. (iv) LX2 cells were seeded in 6-well plates, and were incubated with different concentrations of Rub-PROTAC or Rub for 10 h, and then were collected by RIPA solution for detection by western blot assay. (ⅶ) LX2, JS1, AML12, iBMDM and HHSEC cells were seeded in each 3 cm culture dishes, respectively. LX2 and JS1 cells were activated with TGF-β1 (10 ng/mL), iBMDM cells were activated with lipopolysaccharide (LPS, 100 ng/mL), and HHSEC cells were activated with vascular endothelial growth factor (VEGF, 10 ng/mL), AML12 cells did not require stimulation. All cells were simultaneously treated with Rub at 150 μM for 48 h, then harvested for CPT1A activity assay.

### CCK8 cell viability assay

LX2, JS1, and AML12 cells were seeded into 96-well plates with 100 μL at 5×10^3^ cells per well. When the cells grow to 70-80%, they were incubated with different concentrations of Rub or Erastin or Ferrostatin-1 (Fer-1). Subsequently, the cells were cultured for a specified duration (48 h), after which 10 μL of CCK-8 solution was added to each well, followed by an additional 2 h of incubation. Finally, cell viability was assessed by measuring the absorbance at 450 nm using a microplate reader.

### CPT1A knockdown and site mutation in LX2 cells

To achieve knockdown of human CPT1A in LX2 cells, pLV2N-U6-Puro vector (GenePharma Co., Ltd., Shanghai, China) was utilized to insert control (shNC) or CPT1A-targeting shRNA (shCPT1A-1 and shCPT1A-2) templates. TGF-β1-activated LX2 cells were transfected with the above shRNA to knock down CPT1A expression using Lipofectamine 3000. The shRNA sequences were as follows: shNC: TTCTCCGAACGTGTCACGT, shCPT1A-1: GGATGGGTATGGTCAAGATCT, shCPT1A-2: GGATCTGCTGTATATCCTTCC. In addition, for the CPT1A site-directed mutagenesis experiment, TGF-β1 activated-LX2 cells with shRNA knockdown of endogenous CPT1A were transfected with plasmids expressing of wild-type CPT1A or individual disruptive mutants using Lipofectamine 3000.

### Immunofluorescence assay

LX2 and JS1 cells were seeded into 48-well plates, activated with TGF-β1 (10 ng/mL) and were simultaneously treated with different concentrations of Rub (50, 100 and 150 μM) or SB-431542 (a TGF-β1 inhibitor, 10 μM). After 48 h, the cells were sequentially fixed with 4% paraformaldehyde for 15 min, permeabilized with 0.5% Triton X-100 for 10 min, blocked with 10% goat serum for 30 min, and incubated overnight at 4 ℃ with anti-α-SMA and anti-Col1A1 antibodies. In addition, frozen sections of liver tissue were cut into 8 μM slices, allowed to stand at room temperature for 10 min, fixed with pre-cooled acetone for 15 min, blocked with 10% goat serum for 30 min, and incubated at 4 ℃ for anti-α-SMA, anti-CD11b, anti-CD34, anti-GPX4 and anti-ACSL4 antibodies overnight. The next day, after washing with PBS, the samples were incubated with fluorescent secondary antibodies at room temperature for 30 min, nuclei were stained with DAPI for 1 min, and finally the samples were observed under a fluorescence microscope.

### Cellular biochemical indicators related to ferroptosis assays

LX2, JS1 and AML12 cells were cultured into culture plates, LX2 and JS1 cells were activated with TGF-β1 (10 ng/mL), the cells were simultaneously treated with different concentrations of Rub (50, 100 and 150 μM) or Erastin (40 μM). After 48 h, the cells were separately incubated with Lipid Peroxide, (LPO)-BODIPY 581/591 C11 staining solution, Reactive Oxygen Species (ROS)-DCFH-DA probe solution and Fe^2+^ probe solution for 1 h. And then the cells were washed three times with PBS and observed with microscope. In addition, the cells were also collected using NADP^+^/NADPH or GSH/GSSG extraction solution, and then added with corresponding reagents to detect NADPH, GSH and GSSG content according to the manufacturers'instructions of the reagent kit.

### CPT1A activity assay

Mitochondrial fractions were isolated from whole-cell lysates of LX2, JS1, AML12, HHSEC, and iBMDM cells, and CPT1A activity was measured using a CPT1 activity assay kit in accordance with the manufacturer's instructions.

### RNA isolation and quantitative real-time polymerase chain reaction (qPCR) assay

Total RNA from the cells and the liver tissues was extracted using an Ultrapure RNA kit. Briefly, the liver tissues (30 mg) were homogenized and the cells were lysed with TRIzol reagent, the homogenized tissue solution and lysed cell solution were sequentially added with TRIzon PaI™, 75% ethanol, Buffer RW1, Buffer RW2 and RNase-Free Water, and then total RNA were dissolved using RNase-Free Water. Next, total RNA concentrations were measured and reverse-transcribed into cDNA using HiFiScript All-in-one RT Master Mix for qPCR analysis. The relative mRNA expression levels in the liver tissues and the cells were detected using SuperStar Universal SYBR Master Mix with the ABI ViiA7 sequence detector (Applied Biosystems, USA). The relative expression of genes was analyzed by the 2^-∆∆Ct^ method, GAPDH was used as an internal reference gene. The primer sequences were shown in Supplementary materials Table S1.

### Western blot assay

The protein from the cells and the liver tissues was extracted using RIPA lysis buffer with proteinase inhibitor, and the concentrations of protein lysates were detected using BCA protein assay kit. Next, the protein samples (30 μg) were prepared, separated and transferred onto polyvinylidene difluoride (PVDF) membranes. The PVDF membranes were incubated with primary antibodies overnight at 4℃ as follows: Anti-α-SMA antibody (1:5000), Anti-CPT1A antibody (1:1000), Anti-ACSL4 antibody (1:1000), Anti-GPX4 antibody (1:1000), Anti-NRF2 antibody (1:1000), Anti-c-Myc antibody (1:1000), Anti-Histone H3 antibody (1:1000), Anti-Ubiquitin antibody (1:1000), Anti-GAPDH antibody (1:5000). The next day, the PVDF membranes were washed with PBST and incubated with HRP-conjugated Goat Anti-Rabbit IgG(H+L) or HRP-conjugated Goat Anti-Mouse IgG(H+L) (1:5000), the protein bands on PVDF membranes were visualized by Omni ECL™ Pico Light Chemiluminescence liquid using Invitrogen iBright 1500 imaging system (Thermo Fisher Scientific Co., Ltd., Massachusetts, USA).The visualized bands were quantified using ImageJ software (National Institutes of Health, Bethesda, MD, USA).

### Cellular transmission electron microscopy (TEM) assay

LX2, JS1 and AML12 cells were seeded in 3 cm culture dishes. After drug treatment, the cells were washed with PBS and sequentially fixed with 2.5% glutaraldehyde and 1% osmic acid solution, and then the samples were dehydrated, infiltrated, embedded, sliced and stained. Finally, the ultrastructural changes of cell mitochondria were observed using H-7650B Transmission electron microscope (Hitachi, Tokyo, Japan).

### Statistical analysis

All data were expressed as mean ± standard deviation (SD). Statistical analyses were performed using the SPSS 21.0 software package. Normally distributed and variance homogeneous data were analyzed using one-way analysis of variance (ANOVA) followed by the post hoc least significant difference (LSD) multiple comparisons procedure. Non-normally distributed and unequal variance data were analyzed using the Kruskal-Wallis test. A value of *P* < 0.05 was considered statistically significant.

## Results

### Rub inhibits the activation of HSCs *in vitro*

**Figure 1:**
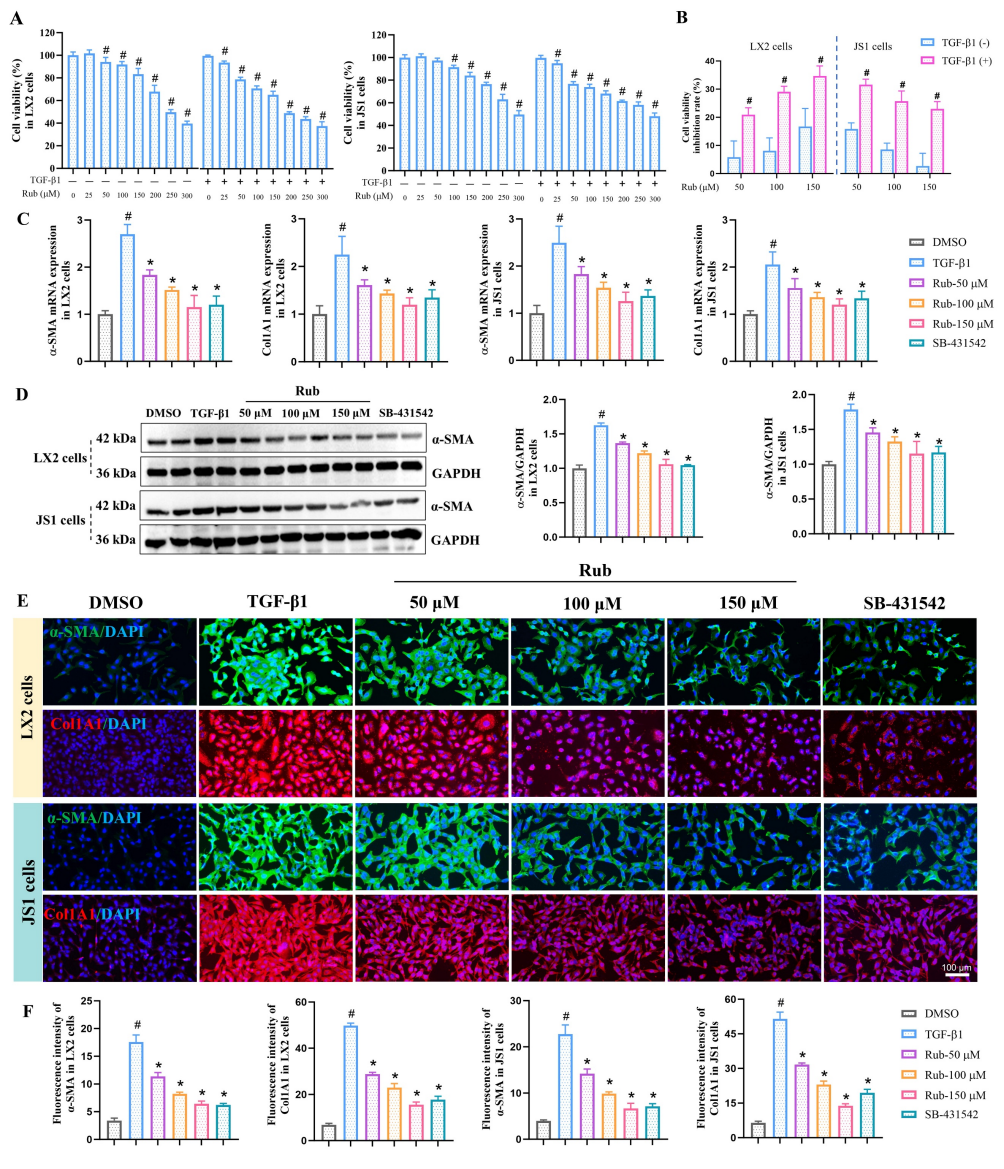
** The effect of Rub on the activation of HSCs in LX2 and JS1 cells.** (A) Cell viability of Rub in LX2 and JS1 cells. (B) Cell viability inhibition rate of Rub in LX2 and JS1 cells. (C) The mRNA expressions of α-SMA and Col1A1 in LX2 and JS1 cells. (D) The protein expression of α-SMA in LX2 and JS1 cells. (E) Immunofluorescence staining of α-SMA (green, scale bar = 100 μm) and Col1A1 (red, scale bar = 100 μm) in LX2 and JS1 cells. (F) The fluorescence intensity of α-SMA and Col1A1 positive staining in LX2 and JS1 cells. All experiments were performed with n = 3 independent biological replicates. ^#^*p* < 0.05 *vs* DMSO ; ^*^p < 0.05 *vs* TGF-β1.

To evaluate the inhibitory effect of Rub on HSCs activation* in vitro*, the activation of human HSC line LX2 and mouse HSC line JS1 cells was induced with TGF-β1. Before evaluating the activation of HSCs, the effect of Rub on the cell viability in LX2 and JS1 cells was first evaluated by CCK8 assay. LX2 and JS1 cells were induced with or without TGF-β1 and were simultaneously treated with different concentrations of Rub for 48 h. As displayed in Figure 1A, Rub exhibited the concentration-dependent inhibitory effects on the cell viability of LX2 and JS1 cells to varying degrees at the concentrations ranging from 25 to 300 μM. Obviously, Rub had a more significant inhibitory effect on the cell viability in TGF-β1-induced cells. The cell viability inhibition rate was further analyzed, and it was found that Rub had an inhibition rate of over 20% on LX2 and JS1 cells stimulated by TGF-β1 (range: 20.94-31.63%), while the inhibition rate on LX2 and JS1 cells without TGF-b1 stimulation was lower than 20% (range: 2.70-16.68%), and there was a statistically significant difference between the TGF-β1-stimulated and non-stimulated groups for both cell lines (Figure 1B). Therefore, Rub does not alter the cell viability of quiescent HSCs at concentrations of 50, 100, and 150 μM. Subsequently, the inhibitory effect of Rub on HSCs activation was assessed at the concentration of 50, 100 and 150 μM. LX2 and JS1 cells activation was induced by TGF-β1 and incubated with Rub, and HSCs activation indicator α-SMA and Col1A1 were detected using qPCR and western blot assay. The results showed that the mRNA expressions of α-SMA and Col1A1, and the protein expression of α-SMA in LX2 and JS1 cells were significantly reduced by Rub, exhibiting concentration-dependent inhibition (Figure 1C-D). In addition, the expressions of α-SMA and Col1A1 were also assessed by immunofluorescence assay, and immunofluorescence results showed that the fluorescence intensity of α-SMA and Col1A1 positive staining were significantly enhanced in TGF-β1-induced LX2 and JS1 cells, but remarkably inhibited by Rub at different incubation concentrations (Figure 1E-F). Altogether, these results confirmed that Rub is a potential inhibitor that can significantly inhibit the activation of HSCs.

### Rub effectively ameliorates liver injury and fibrosis in mice induced by CCl_4_

**Figure 2:**
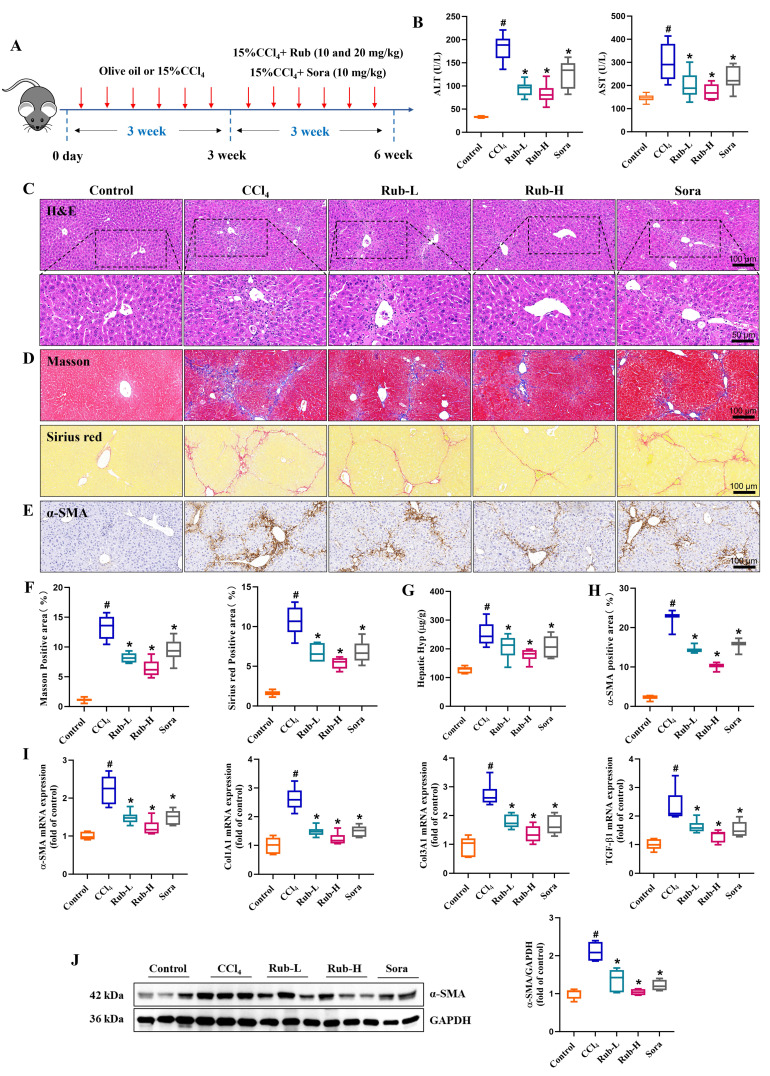
**The effect of Rub on liver injury and fibrosis in CCl_4_-induced mice.** (A) Scheme of the experimental flow for mice injected with CCl_4_ and treated with Rub or Sora. (B) The serum activities of ALT and AST. (C) H&E staining (scale bar = 100 μm and 50 μm). (D, F) Masson and Sirius red staining (scale bar = 100 μm), and its semiquantitative analysis. (E, H) Immunohistochemistry staining of α-SMA (yellow brown, scale bar = 100 μm), and its semiquantitative analysis. (G) Hepatic Hyp conten. (I) The mRNA expressions of α-SMA, Col1A1, Col3A1 and TGF-β1. (J) The protein expression of α-SMA. n = 6,^ #^*p* < 0.05 *vs* Control ; ^*^p < 0.05 *vs* CCl_4_.

To examine the pharmacological effects of Rub on the liver injury and fibrosis, a mouse liver fibrosis model was established by intraperitoneal injection of 15% CCl_4_ thrice weekly for 6 weeks, and Rub (10 and 20 mg/kg) was administered by gavage starting from the third week (Figure 2A). After the experiment, the liver injury and fibrosis indicators in mice were assessed one after another. The elevated serum liver injury markers in CCl_4_ group, such as AST and ALT levels, were significantly reduced by Rub treatment (Figure 2B). Meanwhile, H&E staining results of liver histopathology were shown in Figure 2C, compared with the control group, the liver tissue of CCl_4_-induced liver fibrosis mice showed obvious histological changes, the normal lobular structure was disrupted, hepatocytes swelled and had vacuolar degeneration with enlarged, sinusoidal spaces narrowed and fibrous septa widened, prominent inflammatory cell infiltration in the liver tissue. However, these pathological changes were significantly altered by Rub treatment. In addition, the deposition of collagen fibers was evaluated by Masson staining and Sirius red staining. In the CCl_4_-induced liver fibrosis mice, the collagen deposition was prominent, fibrous septa stained intensely, showing thickening and extensive distribution. Moreover, Masson-positive areas and Sirius red-positive areas were significantly elevated. After Rub treatment, the deposition of collagen fibers in fibrotic mice was markedly alleviated (Figure 2D and F). At the same time, a vital indicator of collagen metabolism, hepatic Hyp content, was also detected and the result showed that the Hyp content was evidently decreased after treatment with Rub (Figure 2G).

HSCs activation is commonly used to evaluate the occurrence and development of hepatic fibrosis *in vivo*. Therefore, in this work, HSCs activation related indicators were also evaluated by qPCR, western blot, and immunohistochemistry assay, just like *in vitro* experiments. The results were shown in Figure 2E and H, immunohistochemistry results showed that α-SMA positive staining were obviously increased in the CCl_4_ group and were significantly decreased in the Rub group. The results of qPCR and western blot assay showed that the elevated expression levels of α-SMA and Col1A1 mRNA, and α-SMA protein in the liver tissues with CCl_4_-induced mice were markedly decreased by Rub treatment (Figure 2I-J). Furthermore, some fibrosis-related gene Col1A3 and TGF-β1 mRNA expression levels in the CCl_4_ group were also significantly reduced by Rub treatment (Figure 2I). These results suggested that Rub could inhibit the activation of HSCs induced by CCl_4_ in mice. Notably, its therapeutic efficacy was comparable to or even superior to that of the positive control Sora across all the tested parameters. Collectively, these data showed that Rub effectively ameliorates liver injury and fibrosis in mice induced with CCl_4_.

### Rub regulates the ferroptosis pathway in RNA-seq analysis

**Figure 3:**
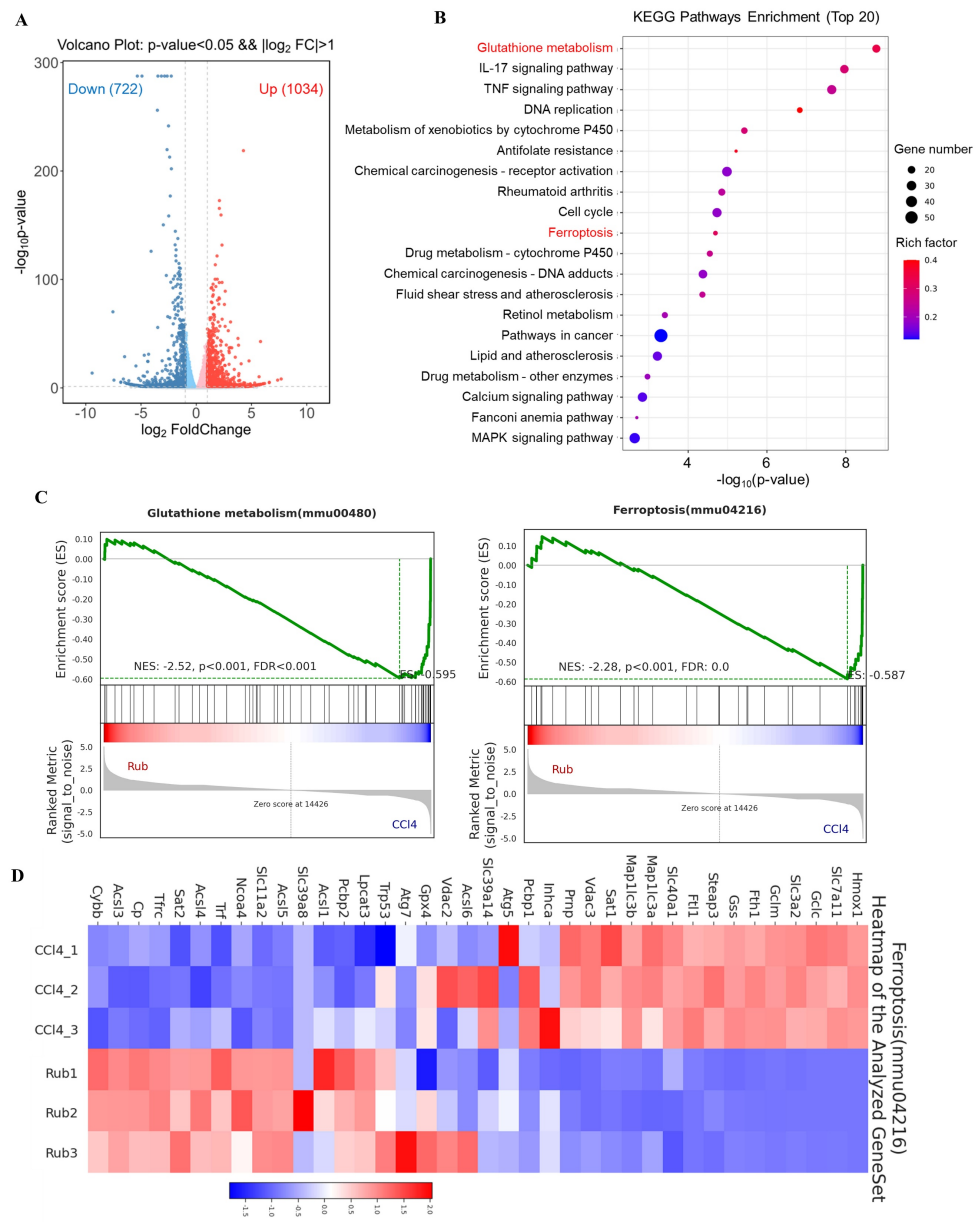
** The effect of Rub on the ferroptosis pathways based on RNA-seq analysis.** (A) Volcano plot showing the log2-fold change of mRNA transcript levels. (B) KEGG pathways enrichment analysis (top 20). (C) GSEA analysis of Glutathione metabolism and Ferroptosis pathways. (D) Heatmap of Ferroptosis pathway-related gene expression profiles based on GSEA analysis.

To explore the molecular mechanism underlying the anti-liver fibrosis effect of Rub, RNA-seq analysis was performed on fibrotic liver tissue treated with or without Rub. Volcano plot analysis of the differentially expressed genes revealed global changes in gene expression patterns, a total of 722 down-regulated genes and 1034 up-regulated genes were enriched in the Rub-vs-CCl_4_ group (Figure 3A). In KEGG pathway analysis, Glutathione metabolism and Ferroptosis pathways were significantly enriched (Figure 3B). Furthermore, these two pathways were further analyzed by Gene set enrichment analysis (GSEA), and the values of NES,* P*, and FDR were as follows: Glutathione metabolism (NES = -2.52, *p* < 0.001, FDR < 0.001) and Ferroptosis (NES = -2.28, *P* < 0.001, FDR = 0.0) (Figure 3C), suggesting that these two pathways were also significantly enriched in GSEA. Glutamate metabolism participates in ferroptosis and is tightly linked to this process. A heatmap of the differentially expressed genes in Ferroptosis pathway was shown in Figure 3D; for instance, the expressions of Hmox1, Slc7a11 and Gclc were significantly decreased, whereas the expressions of Acsl3, Acsl4 and Acsl5 were significantly increased by Rub treatment. Altogether, Rub significantly induces ferroptosis in liver fibrosis, which may be a potential molecular mechanism for Rub's anti-liver fibrosis effect.

### Rub induces the ferroptosis of activated HSCs

**Figure 4:**
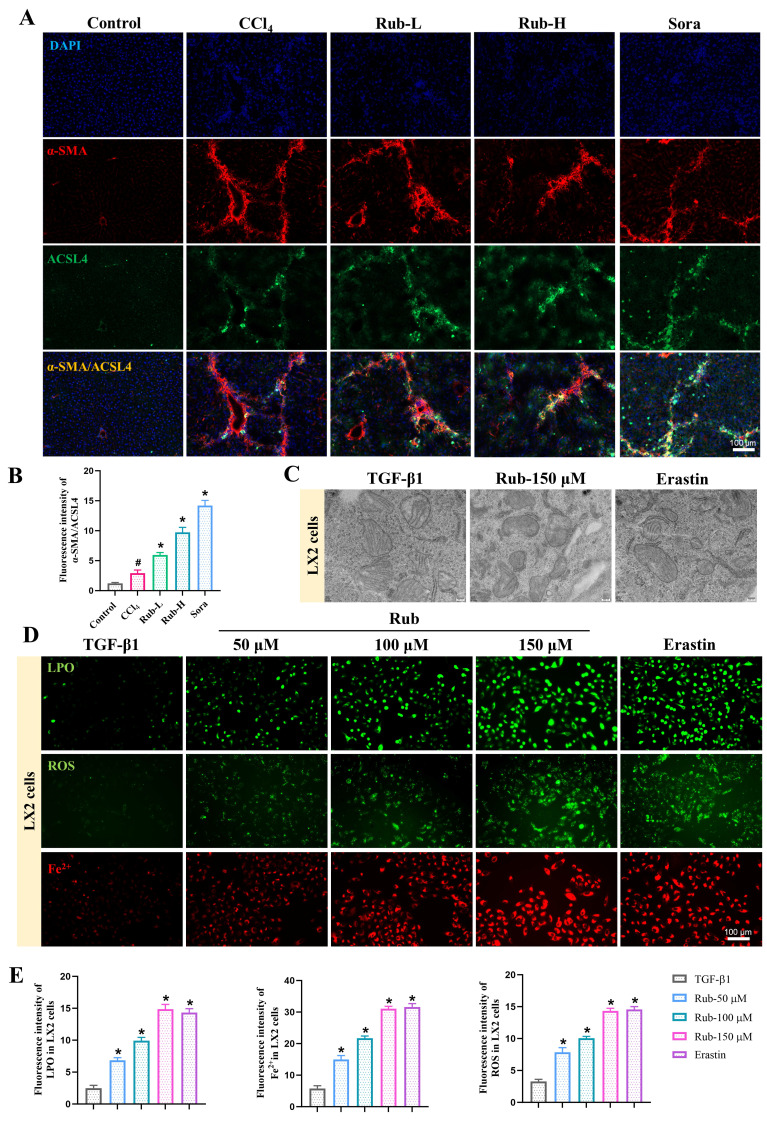
**The effect of Rub on the ferroptosis of activated HSC.** (A) Immunofluorescence co-staining of ACSL4 (green) and α-SMA (red) in CCl_4_-induced mice liver sections (scale bar = 100 μm). (B) Semiquantitative analysis of ACSL4 and α-SMA co-staining. (C) The changes of mitochondrial ultrastructure in TGF-β1-induced LX2 cells (scale bar = 200 nm). (D, E) Fluorescence staining of LPO, ROS, and Fe^2+^ in TGF-β1-induced LX2 cells (scale bar = 100 μm). All experiments were performed with n = 3 independent biological replicates. ^#^*p* < 0.05 *vs* Control; ^*^p < 0.05 *vs* CCl_4_ or TGF-β1.

To determine the specific type of liver cells in which Rub-induced ferroptosis, we evaluated the effects of Rub on ferroptosis in HSCs, macrophages, sinusoidal endothelial cells, and hepatocytes. Considering that HSCs is the key effector cells mediating liver fibrosis, and emerging evidence has shown that inducing the ferroptosis of activated HSCs is an effective measure for treating liver fibrosis 20, we first evaluated whether Rub induced the ferroptosis of activated HSCs. In our work, Rub-induced ferroptosis of activated HSCs was observed in Rub-treated CCl_4_-induced mice. Specifically, immunofluorescence staining results showed that the co-staining of a positive regulator of the ferroptosis process (ACSL4) and an activated HSCs marker (α-SMA) was significantly up-regulated in Rub-treated fibrotic mouse (Figure 4A and B). Subsequently, we conducted a series of experiments to evaluate the effect of Rub on the ferroptosis of activated HSCs *in vitro*. Firstly, TGF-β1-induced LX2 and JS1 cells were incubated together with Rub and Fer-1 (a ferroptosis inhibitor), and the results indicated that the inhibitory effect of Rub on the cell viability could be eliminated by Fer-1 (Supporting materials Figure S1A). It has been confirmed that during ferroptosis, the morphological changes of cell mitochondria are manifested as a reduction in mitochondrial volume, an increase in the density of the double membrane, a reduction or disappearance of mitochondrial cristae 18. The results were shown in Figure 4C and Supporting materials Figure S1B), the mitochondrial morphological alterations associated with ferroptosis were observed in Rub-treated activated LX2 and JS1 cells. Meanwhile, the changes of other important indicators of ferroptosis were also found in Rub-treated activated LX2 and JS1 cells, which were evidenced by decreased the content of GSH, increased the levels of GSSG, LPO, ROS and Fe^2+^ (Figure 4D-E and Supporting materials Figure S1C-D). Additionally, the protein level of GPX4 were markedly downregulated, and the protein level of ACSL4 was significantly upregulated by Rub treatment (Supporting materials Figure S1E). In addition, we also evaluated the effect of Rub on apoptosis or necrosis of HSCs (the two most common alternative cell death modes), and the results showed that Rub did not significantly regulate these two types of cell death in HSCs (Supporting materials Figure S2). These results indicated that Rub induces ferroptosis in HSCs.

From the immunofluorescence results of ferroptosis key indicator ACSL4, it can be concluded that in liver fibrosis, Rub regulates the expression of these proteins mainly in the interstitial cells of the fibrous septum, indicating that Rub induced ferroptosis in liver cells is mainly in the interstitial cells. Therefore, we further evaluated the effect of Rub on ferroptosis of macrophages and hepatic sinusoidal endothelial cells. The results showed that the Rub treatment group had very little immunofluorescence co-staining of ACSL4 with CD11b (hepatic Kupffer markers) and CD34 (hepatic sinusoidal endothelial cells), and there was no significant difference compared to the CCl_4_ model group (Supporting materials Figure S3A-B). In addition, we also evaluated the effect of Rub on the ferroptosis of hepatocytes *in vitro*, and the results showed that Rub did not promote ferroptosis in hepatocytes line AML12 cells (Supporting materials Figure S4). In conclusion, above results confirmed that Rub induces the ferroptosis of activated HSCs in liver fibrosis.

### CPT1A is a degradation target of Rub-PROTAC

**Figure 5:**
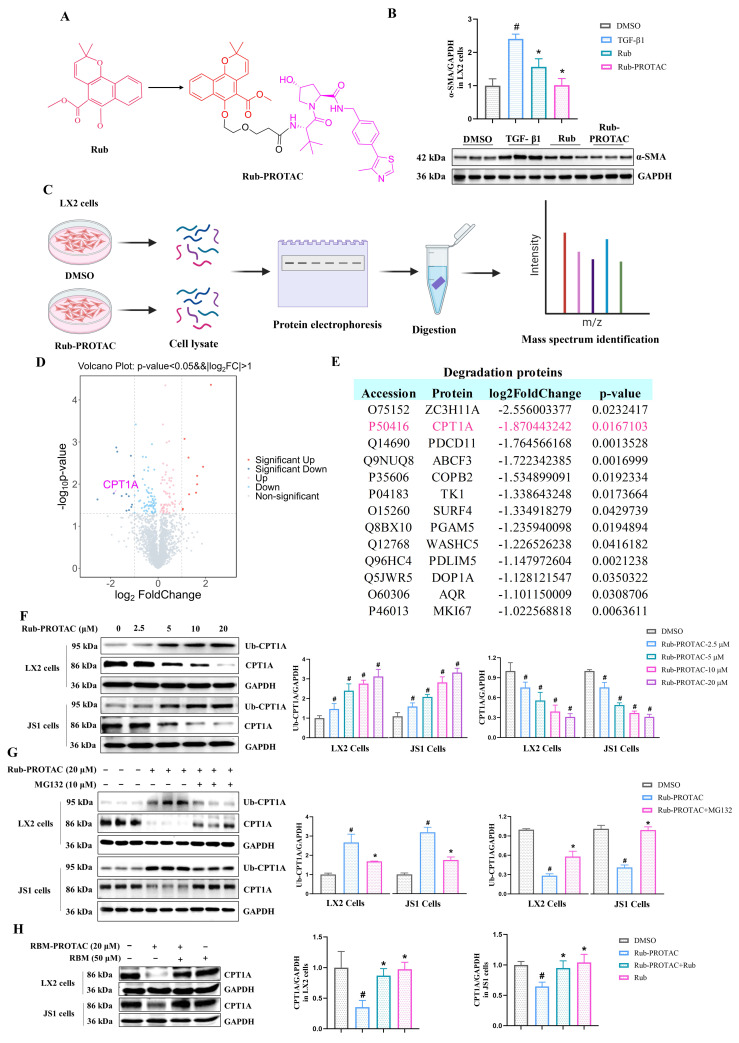
** Analysis and determination of Rub target proteins in LX2 cells using PROTAC technology.** (A) The synthesize of Rub-probe with a PROTAC molecules (Rub-PROTAC). (B) The protein exprssion of α-SMA in LX2 cells treated with Rub and Rub-PROTAC. (C) Procedure of target identification by 4D-DIA proteomics analysis. (D) Differential protein volcano diagram. (E) Degradation proteins with log2FoldChange < -1. (F) The concentration-dependent ubiquitination and degradation of CPT1A protein in Rub-PROTAC-treated LX2 and JS1 cells. (G) The ubiquitination and degradation of CPT1A protein in MG132-and Rub-PROTAC-treated LX2 and JS1 cells. (H) The protein level of CPT1A in LX2 and JS1 cells treated by Rub-PROTAC together with Rub for 10 h. All experiments were performed with n = 3 independent biological replicates. ^#^*p* < 0.05 *vs* DMSO; ^*^p < 0.05 *vs* TGF-β1 or Rub-PROTAC.

In order to investigate the target of Rub in anti-liver fibrosis, we used PROTAC target-fishing technology to identify its target in LX2 cells. Firstly, Rub was selected to synthesize a PROTAC molecule. To synthesize the PROTAC of Rub, the core scaffold structure of the most active Rub was selected as the POI ligand. We coupled Rub with a 1-polyethylene glycol (1-PEG) linker conjugated to thalidomide and obtained a PROTAC molecule (referred to as Rub-PROTAC) (Figure 5A and Supporting materials Scheme S1). Meanwhile, Rub-PROTAC also showed significant inhibitory activity on the protein expression of α-SMA in TGF-β1-stimulated LX2 cells (Figure 5B). To study direct rather than indirect protein degradation, LX2 cells were treated with Rub-PROTAC and DMSO for only 10 h; and 4D-DIA proteomics was used to analyze the proteins significantly degraded by Rub-PROTAC (Figure 5C). As shown in Figure 5D and E, some proteins were significantly down-regulated, such as ZC3H11A, CPT1A, PDCD11, ABCF3 and COPB2. Among these proteins, studies have reported that CPT1A is essential for the development of liver fibrosis and the process of ferroptosis 24,25. In liver fibrosis, loss of CPT1A was found to alleviate HSCs activation and liver fibrosis 24. Furthermore, it has been shown that CPT1A deficiency can induce ferroptosis in lung cancer cells 25. Combining the analysis results with the pharmacological effects of Rub, we ultimately focused on CPT1A in our study. To validate the results from proteomic analysis, LX2 and JS1 cells were incubated with different concentrations of Rub-PROTAC for 10 h, the results showed that CPT1A ubiquitination (Ub-CPT1A) was concentration-dependently enhanced, whereas CPT1A protein was concentration-dependently degraded by Rub-PROTAC (Figure 5F). Subsequently, we co-incubated the Rub-PROTAC-treated cells with the ubiquitination inhibitor MG132, and the results showed that Rub-PROTAC-mediated enhancement of Ub-CPT1A and degradation of CPT1A protein could be abrogated by MG132 (Figure 5G). Further, we carried out simultaneous co-incubation of Rub and Rub-PROTAC in LX2 and JS1 cells to evaluate whether there was competitive binding to CPT1A. The results showed that the degradation of CPT1A protein by Rub-PROTAC can be partially reversed by Rub (Figure 5H). In summary, the above results confirmed that Rub-PROTAC degrades CPT1A protein through the ubiquitination pathway, and CPT1A may be a binding target of Rub.

### Rub directly targets to CPT1A in HSCs

**Figure 6:**
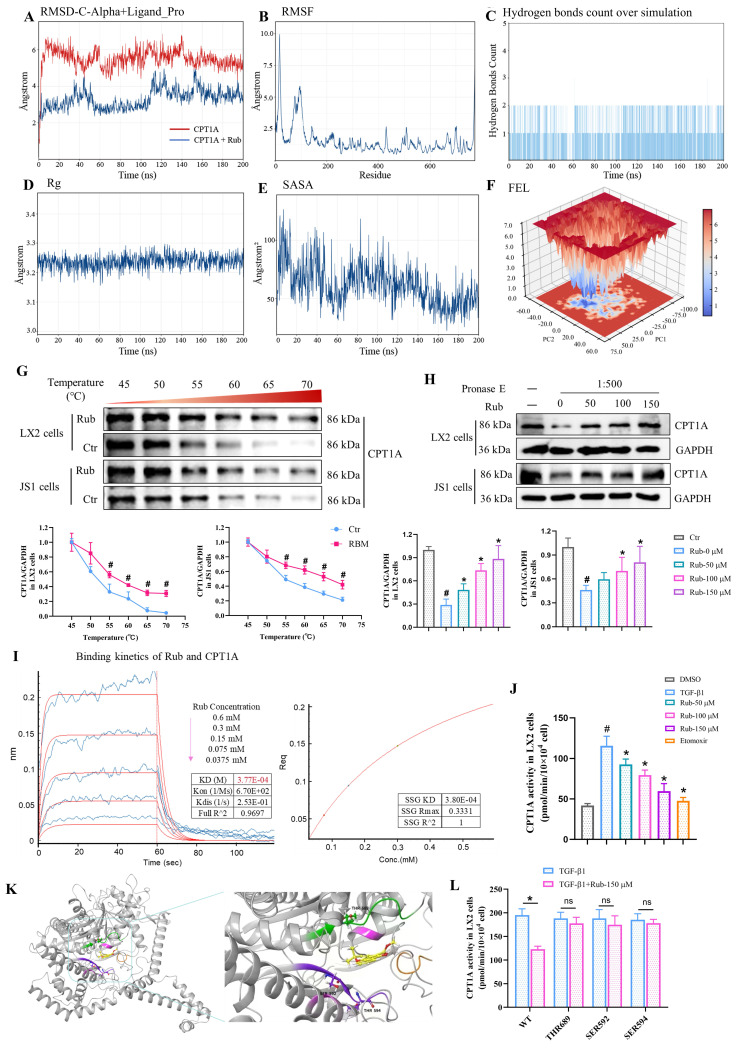
** Verification of Rub directly targets to CPT1A.** (A) The stability of the Rub-CPT1A binding using root-mean-square deviation (RMSD), (B) root-mean-square fluctuation (RMSF), (C) hydrogen bonds count over simulation, (D) radius of rotation (Rg), and (E) solvent accessible surface areas (SASA), (F) free energy landscape (FEL) analysis. (G, H) The protein level of CPT1A in LX2 and JS1 cells using CETSA and DARTS assay. (I) Binding kinetics of Rub and CPT1A in BLI assay. (J) The effect of Rub on CPT1A activity in LX2 cells. (K) Molecular docking simulation in between Rub and CPT1A. (L) The effect of Rub on CPT1A activity in LX2 cells with site mutation. All experiments were performed with n = 3 independent biological replicates. ^#^*p* < 0.05 *vs* Ctr or DMSO ; ^*^p < 0.05 *vs* TGF-β1 or Rub-PROTAC.

To confirm that CPT1A is a direct target of Rub, CPT1A-Rub complex and CPT1A were first selected for molecular dynamics simulation to evaluate the binding of Rub to CPT1A. During the entire simulation process (200 ns), the root-mean-square deviation (RMSD) of the Cα backbone of CPT1A was basically stable at 3 Å, indicating that the system reached an equilibrium state during this simulation process. The RMSD of the Cα backbone of the CPT1A-Rub complex increased from the initial stage of the simulation to 6 Å, then drops back to 5 Å in the middle of the simulation, and remained stably below 5 Å in the later stage of the simulation, indicating that this binding conformation has high thermodynamic stability (Figure 6A). From the RMSF results, it could be seen that the root mean square fluctuations (RMSF) of the amino acid residues at the active site of CPT1A was basically below 2.0-2.5 Å, indicating that the ligand binding of CPT1A-Rub could stabilize the local conformation of the active site (Figure 6B). At the same time, we also monitored the change in hydrogen bonds, radius of rotation (Rg), and solvent accessible surface areas (SASA) for the CPT1A-Rub system (Figure 6C-E). These results further indicated that Rub bound stably to CPT1A throughout the simulation process; the number of hydrogen bonds in CPT1A-Rub remains stable, and the hydrogen bonding sites were also consistent. Moreover, free energy landscape (FEL) analysis showed that PC1(20)/PC2(-20) was a low-energy depression, suggesting that the conformation of Rub and CPT1A was stable at this time (Figure 6F). These data indicated that Rub can bind stably to CPT1A throughout the simulation process.

Subsequently, cellular thermal shift assay (CETSA), drug affinity responsive target stability (DARTS) assay, and bio-layer interferometry (BLI) assay were used to evaluate the ability of Rub to bind to CPT1A, respectively. In CETSA assay, Rub obviously increased the cellular thermal stability of CPT1A protein in LX2 and JS1 cells (Figure 6G). In DARTS assay, the degradation of CPT1A was significantly reduced after Rub incubation in LX2 and JS1 cells (Figure 6H). In BLI assay, the equilibrium dissociation constant (KD) values of Rub for CPT1A were 3.77E-04 mol/L (Figure 6I). In addition, we evaluated the activity of Rub on CPT1A and found that Rub significantly inhibited CPT1A activity in LX2 and JS1 cells (Figure 6J and Supporting materials Figure S5A). Further, we performed molecular docking simulations to study the binding mode of the Rub-CPT1A complex. The result showed that Rub may form a hydrogen bond network with SER592, THR594 and THR689 of CPT1A (Figure 6K). Finally, SER592, THR594 and THR689 were systematically mutated to alanine in LX2 cells with shRNA-mediated knockdown of endogenous CPT1A, followed by overexpression of wild-type CPT1A, and it was found that these site mutations significantly weakened the inhibition of CPT1A activity by Rub, indicating that SER592, THR594, and THR689 were the key binding sites for Rub to inhibit CPT1A activity (Figure 6L).

To evaluate the specificity of Rub for HSCs over other liver cell types, we detected the CPT1A activity of Rub on TGF-β1-activated LX2, normal murine hepatocytes (AML12), LPS-polarized murine bone marrow-derived macrophages (iBMDM), and VEGF-stimulated human hepatic sinusoidal endothelial cells (HHSEC) at a concentration of 150 μM, and compared the activity inhibition rates between different cells. The results showed that Rub exhibited a significantly higher CPT1A activity inhibition efficiency in LX2 cells than that in AML12, iBMDM, and HHSEC cells (Supporting materials Figure S5B-C). At the same time, we examined CPT1A expression in liver tissue following Rub treatment, and the results showed that Rub significantly reduced CPT1A protein level in CCl_4_-induced liver fibrosis (Supplementary Figure S5D). Altogether, these results suggested that CPT1A is a direct pharmacological target of Rub in HSCs.

### Rub induces ferroptosis *via* targeting CPT1A in activated LX2 cells

Based on previous reports that CPT1A deficiency could alleviate HSCs activation during liver fibrosis, as well as induce ferroptosis in tumor cells, we speculated that CPT1A also plays a role in inducing ferroptosis in activated HSCs. To verify this hypothesis, we performed CPT1A activity inhibitor Etomoxir treatment and CPT1A knockdown experiments to explore the role of CPT1A on inducing ferroptosis in TGF-β1-induced LX2 cells. Firstly, LX2 cells were cultured with different concentrations of Etomoxir for 48 h; the results showed that Etomoxir had no inhibitory effect on the cell viability of LX2 cells without TGF-β1 stimulation, but exerted a significant inhibitory effect on the cell viability of TGF-β1-stimulated LX2 cells at the concentrations of 50 and 100 μM—especially 100 μM, where the cell viability was below 80% (Supplementary Figure S6A). However, Etomoxir-mediated growth inhibition in LX2 cells was abrogated by Fer-1 (Supplementary Figure S6B). Next, ferroptotic mitochondrial morphological alterations were obviously observed; the contents of NADPH and GSH were markedly decreased, and the levels of GSSG, LPO, ROS and Fe^2+^ were significantly increased in Etomoxir-treated LX2 cells with TGF-β1 stimulation (Supplementary Figure S6C-E). Furthermore, ACSL4 protein expression was significantly increased, but GPX4 protein expression was significantly reduced in Etomoxir-treated LX2 cells induced by TGF-β1 (Supplementary Figure S6F). As expected, the results of CPT1A knockdown in TGF-β1-induced LX2 cells were consistent with Etomoxir-treated LX2 cells (Supplementary Figure S6C-F). In addition, Phospholipids are the basic components of the lipid bilayer that makes up the cell membrane, and are classified according to their head groups, including phosphatidylcholine (PC), phosphatidylethanolamine (PE), phosphatidylserine (PS), phosphatidylinositol (PI), and phosphatidic acid (PA) 18,19,40,41. PL-PUFAs, serving as the primary substrates for lipid peroxidation and produced by ACSL4, are considered to be drivers of ferroptosis 19. Thus, a non-targeted lipidomics was performed to analyze the metabolic status of PL-PUFAs, the results showed that increased levels of PL-PUFAs, specifically PC-PUFAs, PE-PUFAs and PI-PUFAs, were observed in shCPT1A-transfected LX2 cells (Supplementary Figure S6G). Altogether, these data suggested that blocking CPT1A induces metabolic reprogramming-mediated the ferroptosis of activated HSCs.

**Figure 7:**
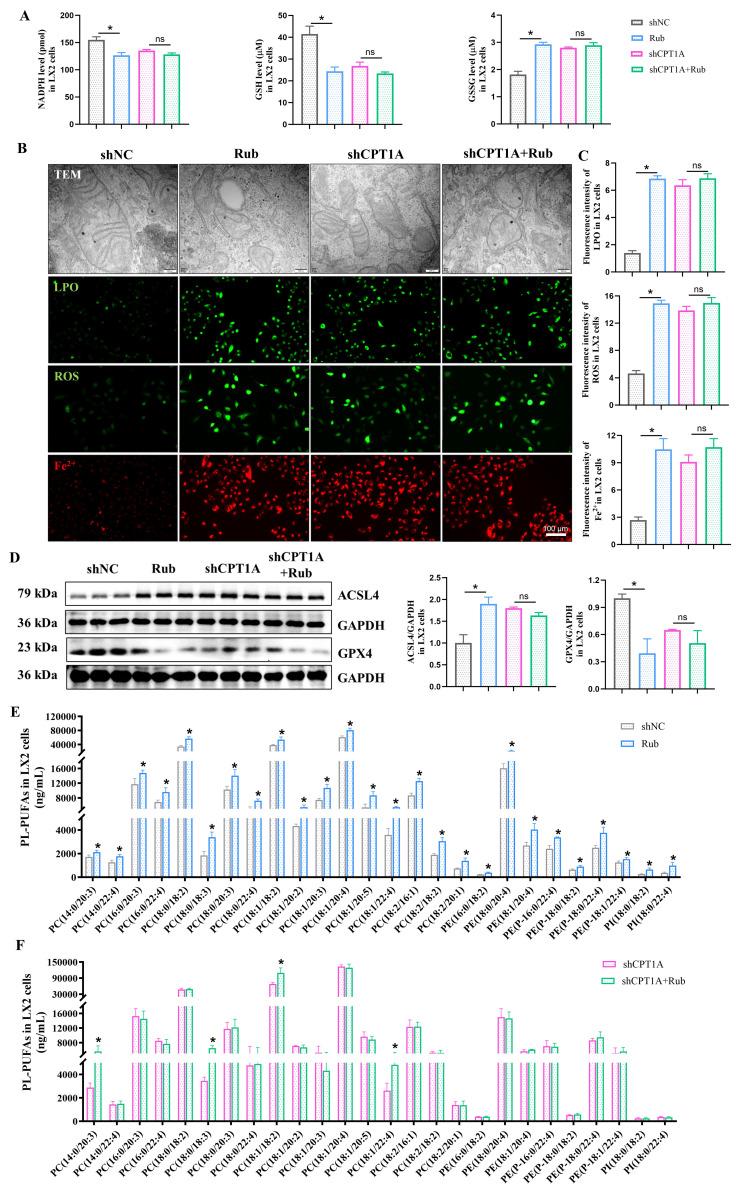
** The effect of CPT1A deficiency on Rub-induced the ferroptosis TGF-β1-induced LX2 cells.** (A) The levels of NADPH, GSH and GSSG in LX2 cells. (B and C) The changes of mitochondrial ultrastructure (scale bar = 200 μm), fluorescence staining of LPO, ROS, and Fe^2+^ (scale bar = 100 μm) in LX2 cells. (D) The protein expressions of GPX4 and ACSL4 in LX2 cells. (E and F) The level changes of PL-PUFAs in LX2 cells. All experiments were performed with n = 3 independent biological replicates.^ *^p < 0.05 *vs* shNC.

To verify that Rub-induced ferroptosis in activated HSCs is mediated by targeting CPT1A, we performed a CPT1A knockdown experiment to explore the role of CPT1A in Rub-induced ferroptosis in TGF-β1-induced LX2 cells. As shown in Figure 7A-E, Rub could obviously induce the changes of the ferroptotic mitochondrial morphology, significantly reduce the contents of NADPH and GSH, elevate the levels of GSSG, LPO, ROS, Fe^2+^ and PL-PUFAs, as well as increase ACSL4 protein expression and inhibit GPX4 protein level in TGF-β1-stimulated LX2 cells. Subsequently, we transfected shRNA against CPT1A into TGF-β1-induced LX2 cells, followed by Rub incubation. The results revealed that remarkable changes in Rub-induced ferroptosis were significantly abolished after the addition of shCPT1A (Figure 7A-F). In other words, Rub did not further aggravate ferroptosis in shCPT1A-transfected LX2 cells. Additionally, the effect of CPT1A overexpression on Rub-induced ferroptosis was also evaluated. Similar to the results of CPT1A knockdown, CPT1A overexpression also abolished Rub's induction of ferroptosis in LX2 cells (Supporting materials Figure S7A-D). These data indicated that both CPT1A-knockdown and CPT1A overexpression eliminates Rub-induced ferroptosis in HSCs. In summary, these results demonstrated that the ferroptosis-inducing effect of Rub depends on CPT1A-mediated metabolic reprogramming.

### Rub regulates the c-Myc ubiquitination-mediated NRF2/GPX4 and ACSL4/PL-PUFAs pathways through targeting

**Figure 8:**
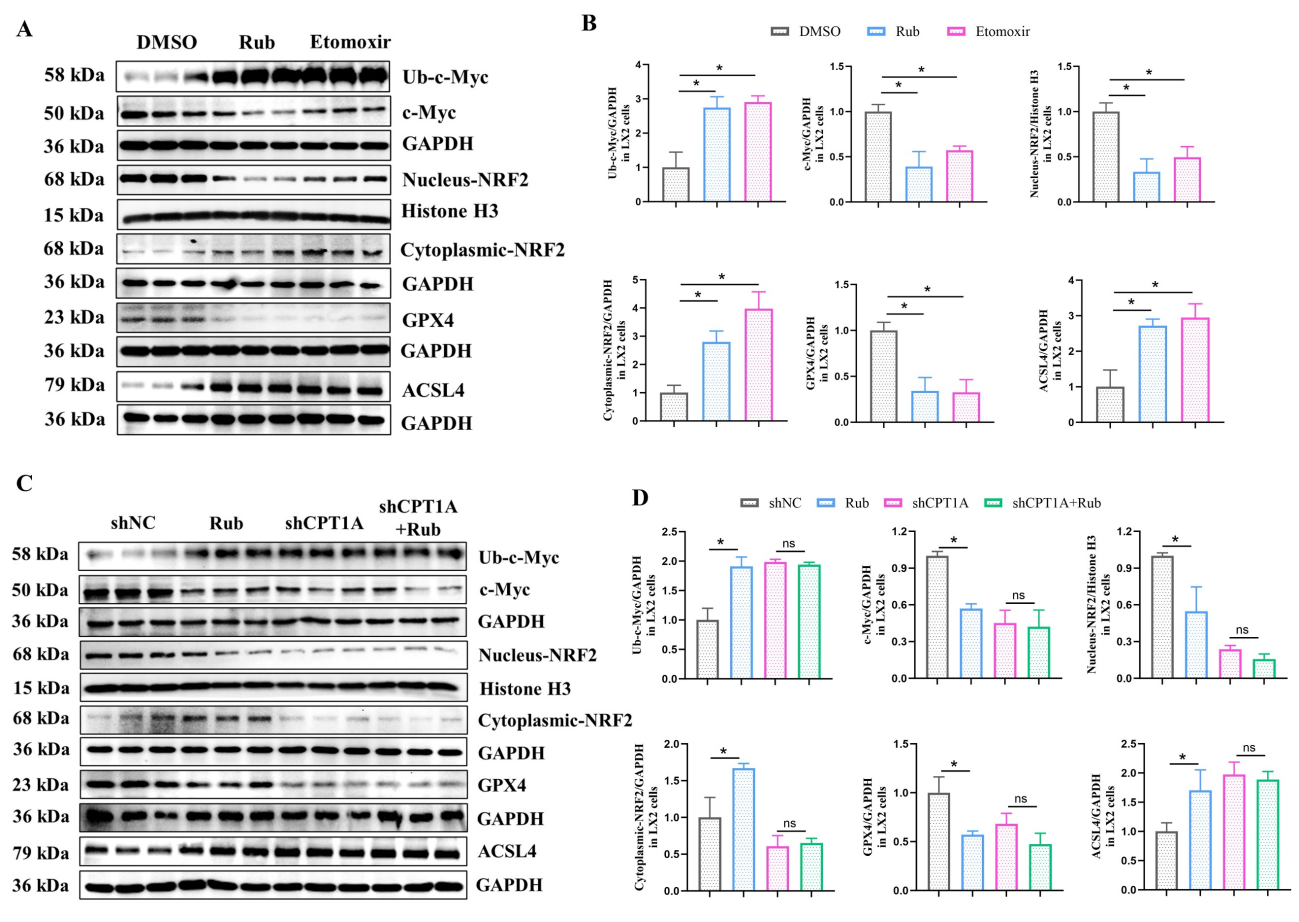
** The effect of Rub on the c-Myc ubiquitination-mediated NRF2/GPX4 and ACSL4/PL-PUFAs pathways in LX2 cells.** (A and B) The protein levels of Rub and Etomoxir on GPX4, ACSL4, Nucleus-NRF2, Cytoplasmic-NRF2, c-Myc ubiquitination and c-Myc in LX2 cells. (C and D) The protein levels of Rub on GPX4, ACSL4, Nucleus-NRF2, Cytoplasmic-NRF2, Ub-c-Myc and c-Myc in LX2 cells. All experiments were performed with n = 3 independent biological replicates. ^*^p < 0.05 *vs* DMSO or shNC.

**Figure 9:**
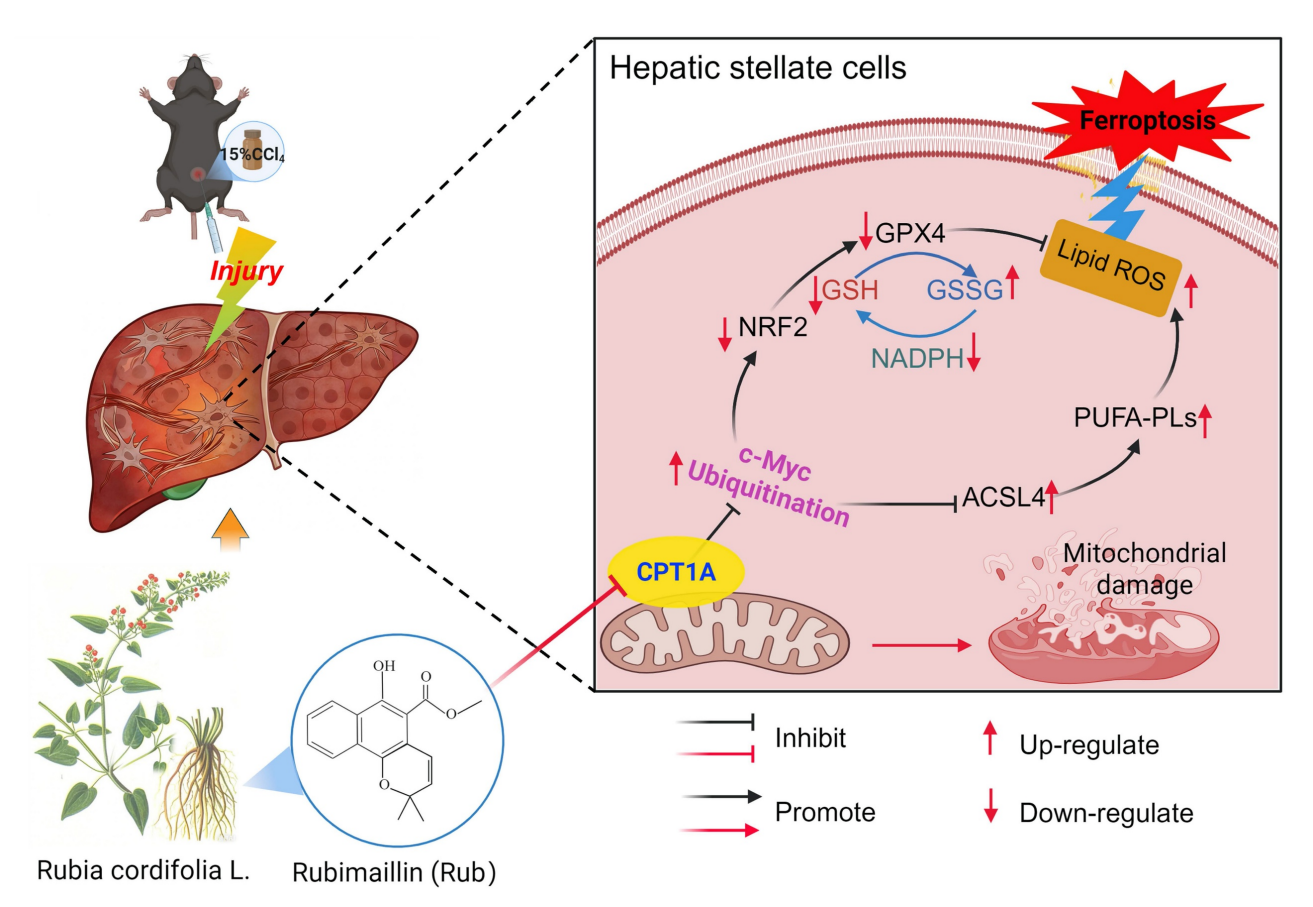
** Rub ameliorates liver fibrosis by inducing metabolic reprogramming-mediated ferroptosis in activated HSCs through targeting CPT1A.** During liver fibrosis, CPT1A was elevated in activated HSCs, which could prevent ferroptosis in activated HSCs through enhancing the NRF2/GPX4 pathway-mediated the antioxidative capacity and inhibiting ACSL4-mediated the production of PL-PUFAs. After treatment, Rub could ameliorate liver fibrosis through inducing metabolic reprogramming-mediated ferroptosis in activated HSCs* via* targeting CPT1A, a process closely linked to c-Myc ubiquitination-mediated regulation of the NRF2/GPX4 and ACSL4/PL-PUFAs pathways.

Next, we continued to explore how Rub induces ferroptosis in HSCs through CPT1A. It has been reported that targeting CPT1A mediates metabolic remodeling to induce ferroptosis in lung cancer stem cells by regulating the NRF2/GPX4 antioxidative system and ACSL4/PL-PUFAs pathway via inhibiting c-Myc ubiquitination 25. Considering the above results of our study, we hypothesized that Rub-induced ferroptosis was tightly correlated with the CPT1A ubiquitination-mediated NRF2/GPX4 and ACSL4/PL-PUFAs pathways in activated HSCs. Therefore, we evaluated the effect of Rub on these pathways. Western blot results showed that the protein levels of GPX4 and nuclear NRF2 were decreased, while the levels of c-Myc ubiquitination (Ub-c-Myc), cytoplasmic NRF2, and ACSL4 protein were elevated (Figure 8A), accompanied by an increase in PL-PUFAs levels in Rub-treated, TGF-β1-induced LX2 cells (Figure 7E). Subsequently, we transfected shRNA against CPT1A into TGF-β1-induced LX2 cells to further investigate the role of CPT1A in Rub-regulated c-Myc ubiquitination, and the NRF2/GPX4 and ACSL4/PL-PUFAs pathways. Western blot results showed that shRNA against CPT1A markedly abolished the ability of Rub to regulate c-Myc ubiquitination, and the NRF2/GPX4 and ACSL4/PL-PUFAs pathways in TGF-β1-induced LX2 cells (Figure 7E, Figure 8C and D). In summary, our results verified that Rub induces metabolic reprogramming-mediated ferroptosis in activated HSCs by regulating the NRF2/GPX4 and ACSL4/PL-PUFAs pathways through facilitating CPT1A-mediated c-Myc ubiquitination (Figure 9).

## Discussion

Liver fibrosis is a complex pathological process characterized by the excessive deposition of ECM components 2. It is widely accepted that activated HSCs are the major effector cells in liver fibrosis, which have a remarkable ability to synthesize and secrete excessive ECM components, especially collagen 11. Active ingredients of traditional Chinese medicine and natural products targeting activated HSCs offer a promising and potentially less-toxic alternative for the treatment of liver fibrosis, holding great potential for the development of novel therapeutic agents in this field 42-44. Rub is an active ingredient in the traditional Chinese medicine *Rubia cordifolia L*. and exhibits various pharmacological activities. In our study, we first evaluated the effects of Rub in a mouse liver fibrosis model induced by CCl_4_, as well as the HSCs activation both *in vivo* and *in vitro*. The results showed that Rub effectively ameliorated liver injury and fibrosis in mice models induced by CCl_4_, and inhibited the activation of HSCs. These results suggested that Rub has great potential as a novel therapeutic agent for liver fibrosis.

Ferroptosis is a recently identified form of regulated cell death, and targeting HSCs ferroptosis has emerged as a potential strategy for treating liver fibrosis 21. Rub was reported to drive ferroptosis to inhibit proliferation in colorectal cancer cells 38. In our study, we first analyzed the liver tissue treated with Rub using RNA-seq assay and found that Rub significantly regulated ferroptosis-related pathways. However, whether it can induce ferroptosis in activated HSCs remains to be studied. To verify the cell-type specificity of Rub-induced ferroptosis, we integrated *in vivo* tissue localization and *in vitro* cellular functional validation to exclude hepatocytes, Kupffer cells, or HSECs, as well as exclude apoptosis or necrosis of HSCs as alternative cell death pathways. Subsequently, we systematically evaluated the effect of Rub on ferroptosis in activated HSCs *in vitro*. The results showed that Rub treatment led to typical ferroptotic changes, including mitochondrial morphological alterations, decreased GSH content, and increased levels of GSSG, LPO, ROS and Fe^2+^, as well as the downregulation of GPX4 protein and the upregulation of ACSL4 protein in LX2 and JS1 cells. These results showed that Rub specifically induces ferroptosis in activated HSCs.

To discover the underlying mechanisms and cellular target proteins of Rub, we designed and synthesized Rub-probe with a PROTAC molecules (Rub-PROTAC). Subsequently, 4D-DIA-based proteomics approach was applied to identify target proteins degraded by Rub-PROTAC. PROTAC technology has previously been used for target identification of TCM-derived natural products (e.g., lathyrane diterpenoids, celastrol, artemisinin, evodiamine, carabrone) 29-33, effectively addressing the drawbacks of conventional techniques. Specifically, it avoids altering the biological activity of natural products via chemical modification and enables in situ target fishing in live cells, thus better mimicking physiological conditions for more reliable target identification. 4D-DIA proteomics results showed that CPT1A protein was degraded efficiently after Rub-PROTAC treatment. Verification results confirmed that the ubiquitination and degradation of CPT1A protein were induced in a concentration-dependent manner by Rub-PROTAC, which were blocked by the ubiquitination inhibitor MG132—confirming that Rub-PROTAC mediates CPT1A degradation through the canonical ubiquitin-proteasome pathway. In addition, Rub-PROTAC-mediated degradation of CPT1A could be competitively inhibited by Rub. Subsequently, molecular dynamics simulation, CETSA, DARST, and BLI assays all strongly confirmed that Rub targets CPT1A. Further site mutation experiment data showed that Rub mainly binds to CPT1A at SER592, THR594 and THR689, indicating that these sites were crucial for Rub to inhibit CPT1A activity. To systematically assess Rub's cell-type specificity for CPT1A, we measured its inhibitory effect on CPT1A activity across four representative cell types of the liver microenvironment, the results showed that Rub inhibited CPT1A activity in all four cell types (HSCs, hepatocytes, macrophages, and HSECs), but exhibited significantly preferential inhibition in HSCs. Based on the above data, we believe that Rub can specifically target CPT1A in activated HSCs.

Previous studies have demonstrated that CPT1A is upregulated in activated HSCs from liver fibrosis models and patients, and this upregulation is a critical driver of HSC activation and subsequent fibrosis development 16. Given that both pharmacological and genetic targeting of CPT1A can suppress HSC activation and alleviate fibrosis 16,17, CPT1A has been proposed as a potential therapeutic target for liver fibrosis 24. Furthermore, CPT1A has been identified as a crucial mediator of ferroptosis resistance, and its targeted inhibition can effectively trigger ferroptosis in lung cancer stem cells 25-27. However, whether CPT1A regulates ferroptosis in activated HSCs remains unknown. Our results found that the inhibition or deficiency of CPT1A induced ferroptosis in activated HSCs, as demonstrated by the experiments with CPT1A activity inhibitor Etomoxir and CPT1A knockdown. Targeting CPT1A could cause mitochondrial morphological alterations, the disturbance of the GSH, GSSG, LPO, ROS, Fe^2+^, and PL-PUFAs homeostasis, and the abnormal expression of GPX4 and ACSL4 proteins to induce metabolic reprogramming, thereby inducing the ferroptosis of activated HSCs in LX2 cells. Although the above results have confirmed that Rub directly binds to CPT1A, as well as both Rub and CPT1A inhibition induced the ferroptosis of activated HSCs, it remains to be clarified whether Rub induced metabolic reprogramming-mediated ferroptosis by targeting CPT1A. Subsequently, we carried out a series of experiments to confirm that CPT1A was an important mediator for Rub-induced ferroptosis in activated HSCs. The results showed that the deficiency or overexpression of CPT1A could significantly abolish Rub-induced metabolic reprogramming and ferroptosis in activated LX2 cells. It is concretely represented by the fact that Rub-caused the mitochondrial morphological alterations, the disturbance of the NADPH, GSH, GSSG, LPO, ROS, Fe^2+^, or PL-PUFAs homeostasis, and the abnormal expression of ACSL4 and GPX4 proteins were significantly abolished by CPT1A knockdown. In summary, these validation experiments confirmed that Rub-mediated HSC ferroptosis was dependent on CPT1A.

At present, research has confirmed that CPT1A resists metabolic reprogramming-mediated ferroptosis through regulating the NRF2/GPX4 and ACSL4/PL-PUFAs pathways by inhibiting c-Myc ubiquitination in tumors 25. Thus, we speculated that Rub may induce ferroptosis in activated HSCs through CPT1A-mediated these two pathways. To gain insight into the mechanism by which Rub induces metabolic reprogramming-mediated ferroptosis in activated HSCs, and thereby inhibits liver fibrosis by targeting CPT1A. In this study, we first evaluated the effect of Rub on this pathway in activated HSCs line LX2 cells. The results indicated that c-Myc ubiquitination was enhanced, the NRF2/GPX4 and ACSL4/PL-PUFAs pathways were significantly regulated by Rub treatment. Further results revealed that the deficiency of CPT1A markedly abolished the ability of Rub to regulate c-Myc ubiquitination, as well as the NRF2/GPX4 and ACSL4/PL-PUFAs pathways. In general, our results confirmed that Rub induces activated HSCs ferroptosis through regulating the NRF2/GPX4 and ACSL4/PL-PUFAs pathways-mediated metabolic reprogramming by targeting CPT1A-mediated c-Myc ubiquitination. By targeting CPT1A, Rub may be able to effectively induce metabolic reprogramming and ferroptosis in activated HSCs, and thereby inhibit liver fibrosis, providing insights into the potential mechanism of Rub against liver fibrosis. However, the anti-hepatic fibrosis effect of Rub targeting CPT1A needs to be further verified in mouse fibrosis model with HSCs-specific CPT1A knockout.

Collectively, in this present study, we have discussed that Rub could effectively ameliorate liver fibrosis *via* inducing metabolic reprogramming-mediated the ferroptosis of activated HSCs *in vivo* and *in vitro*. We identified CPT1A as the target of Rub by PROTAC target-fishing technology, and verified that Rub could directly bind to CPT1A using computer molecular dynamics simulations, CETSA, DARTS, BLI, and site mutation assays. Furthermore, we not only demonstrated that CPT1A was an important regulator for inducing the ferroptosis of activated HSCs, but also confirmed that it was a critical target for Rub-induced ferroptosis. Further results showed that Rub induced metabolic reprogramming-mediated ferroptosis in activated HSCs by targeting CPT1A, which may be related to the c-Myc ubiquitination-mediated the NRF2/GPX4 and ACSL4/PL-PUFAs pathways. However, this study also has certain limitations—particularly regarding the validation of CPT1A-mediated specific targeting of HSCs by Rub, which requires further confirmation using *in vivo* HSC-specific CPT1A knockout models. In summary, Rub can ameliorate hepatic fibrosis by inducing metabolic reprogramming-mediated the ferroptosis of activated HSCs through targeting CPT1A, providing a promising therapeutic strategy for liver fibrosis.

## Supplementary Material

Supplementary methods, figures, and table.
