# Supplementary material for: Rubimaillin ameliorates liver fibrosis by triggering the ferroptosis of activated hepatic stellate cells through targeting CPT1A
Source: Int J Biol Sci. 2026 Jan 22;22(4):2065–84. doi: 10.7150/ijbs.120415 (PMC12905648; doi:10.7150/ijbs.120415)
Supplement: Supplementary file 1 — Supplementary methods, figures, and table. [file ijbsv22p2065s1.pdf]

Supporting materials for

Rubimaillin ameliorates liver fibrosis by inducing the ferroptosis of activated hepatic stellate cells through targeting CPT1A

Dingqi Zhang[a,b]*, Qingxuan Tang[a]*, Xiaoli He[d]*, Chengming Wen[a], Fengfeng Zhou[b], Xia Wei[b], Zikang Wang[b], Jiao Wang[b], Wei Liu[b], Ying Xu[c✉], Yunyao Jiang[a✉], Hang Yin[a✉]

[a]*State Key Laboratory of Membrane Biology, School of Pharmaceutical Sciences, Tsinghua-Peking Center for Life Sciences, Key Laboratory of Bioorganic Phosphorous Chemistry and Chemical Biology (Ministry of Education), Tsinghua University, 30 Shuangqing Road, Beijing 100084, China.*

[b]*Department of pharmacy, The NATCM Third Grade Laboratory of Traditional Chinese Medicine Preparations, Key Laboratory of Liver and Kidney Diseases (Ministry of Education), Shuguang Hospital Affiliated to Shanghai University of Traditional Chinese Medicine, 528 Zhangheng Road, Shanghai 201203, China*

[c]*School of Traditional Chinese Medicine, Shanghai University of Traditional Chinese Medicine, 1200 Cailun Road, Shanghai 201203, China*

[d]*Department of Endocrinology, Yueyang Hospital of Integrated Traditional Chinese and Western Medicine Affiliated to Shanghai University of Traditional Chinese Medicine, 110 Ganhe Road, Shanghai 200437, China*

*These authors contributed equally to this work.

✉Correspondence authors: **Hang yin:** School of Pharmaceutical Sciences, Tsinghua University, Beijing 100084, China (Email: yin_hang@tsinghua.edu.cn). **Yunyao Jiang**: School of Pharmaceutical Sciences, Tsinghua University, Beijing 100084, China (Email: yyjiang@tsinghua.edu.cn). **Ying Xu**: School of Traditional Chinese Medicine, Shanghai University of Traditional Chinese Medicine, Shanghai 201203,

China (Email: xuying.911@163.com).

Materials and Methods

Reagents

Rub ($C_{17}H_{16}O_6$, molecular weight: 284.31, 55481-88-4, purity ≥ 98.0 %) was purchased from Chengdu Alfa Biotechnology Co., Ltd. (Chengdu, China). Sorafenib (Sora; MB1666), obeticolic acid (OCA, MB6084) and cell ferrous iron (Fe^{2+}) fluorometric assay kit (MA0647) were purchased from Meilunbio Co. Ltd. (Dalian, China). Olive oil (69018028) and carbon tetrachloride (CCl_4, 10006464) were provided from Sinopharm Chemical Reagent Co., Ltd. (Shanghai, China). The normal-fat diet (D12450B) and high-fat, high-fructose, high-cholesterol diet (HFHFrC, 40%kcal Fat, 20%kcal Fructose, and 2%Cholest, D09100310) were purchased from Research Diets Co. Ltd. (New Brunswick, NJ, USA). Oil red O Stain kit (D027-4) and Hydroxyproline (Hyp) assay kit (A030-2-1) were obtained from Nanjing Jiancheng Bioengineering Institute (Nanjing, China). Triglyceride (TG) detection kit (R0327) was provided from Zhejiang Dongou Diagnostic Products Co., Ltd. (Wenzhou, China). Erastin (S7242, purity: 99.89%) and Ferrostatin-1 (S7243, purity: 99.98%) were obtained from Selleck Chemicals (Houston, TX, USA). Recombinant human TGF-β1 protein was purchased from ABclonal Biotech Co., Ltd. (Wuhan, China). Reactive oxygen species assay kit (S003S), Lipid peroxidation assay kit with BODIPY 581/591 C11 (S0043S), GSH and GSSG assay kit (S0053), $NADP^+$/NADPH assay kit with WST-8 (S0179), Sodium camptothecin (Y026037), and Annexin V-FITC Apoptosis Detection Kit (C1062M) were purchased from Beyotime Biotechnology Inc. (Shanghai, China). Protease inhibitor cocktail (CW2200S), Ultrapure RNA kit (CW0581S), HiFiScript All-in-one RT Master Mix for qPCR kit (CW3371M) and SuperStar Universal SYBR Master Mix kit (CW3360M) were purchased from Jiangsu Cowin Biotech Co., Ltd. (Taizhou, China). RIPA lysate (PC102), BCA protein assay kit (ZJ101L), Omni ECL™ Pico Light Chemiluminescence liquid (SQ201L) and protein free rapid blocking buffer (PS108P) were obtained from Shanghai Epizyme Biomedical Technology Co., Ltd. (Shanghai, China). CPT1A (Human) recombinant protein (H00001374-P01) was purchased from Abnova (Taipei City, Taiwan). CPT1 activity assay kit (CPT1-1-Y) was purchased from Suzhou Comin Biotechnology Co. Ltd. (Suzhou, China). Anti-α-SMA antibody (ab124964) and Goat Anti-Rabbit IgG H&L (Alexa Fluor® 488) (ab150077) were purchased from Abcam, Inc. (Cambridge, UK). Anti-α-SMA antibody (BF9212) and Anti-Col1A1 antibody (AF7001) was purchased from Affinity Biosciences LTD. (Changzhou, China).

Anti-CPT1A antibody (15184-1-AP), Anti-ACSL4 antibody (22401-1-AP), Anti-GPX4 antibody (30388-1-AP), Anti-NRF2 antibody (16396-1-AP), Anti-c-Myc antibody (10828-1-AP), Anti-Histone H3 antibody (17168-1-AP), Anti-GAPDH antibody (10494-1-AP), HRP-conjugated Goat Anti-Rabbit IgG(H+L) (SA00001-2) and HRP-conjugated Goat Anti-Mouse IgG(H+L) (SA00001-1) were purchased from Proteintech Group, Inc. (Wuhan, China). PVDF membrane (0.45 μm, 0000272041) was acquired from Merck Millipore Ltd. (Darmstadt, Germany). LX2, JS1, AML12, and HHSEC cells were acquired from Department of Liver Diseases, Shuguang Hospital Affiliated to Shanghai University of Traditional Chinese Medicine (Shanghai, China). iBMDM were purchased from Zhejiang Meisen Cell Technology Co., Ltd. (Hangzhou, Zhejiang).

RNA-seq assay

Total RNA from liver tissues were extracted with TRIzol and purified using an isolation kit, followed by quality control; mRNA was enriched with oligo(dT) beads, fragmented, and used for cDNA synthesis, end repair, a-overhang ligation, and PCR amplification to generate libraries, which were then validated and sequenced on an Illumina platform. This sequencing was tested and analyzed by HeYuan Biotechnology Co., Ltd. (Shanghai, China).

Cell apoptosis and necrosis assays

LX2 cells were seeded in 24-well plates and treated with Rub at concentrations of 50, 100, and 150 μM, respectively, with 10 μM camptothecin used as a positive control. After 48 h of incubation, the cells were centrifuged at 1000×g for 5 min. The cells were washed once with PBS. Subsequently, 195 μl of Annexin V-FITC binding buffer was added, followed by 5 μl of Annexin V-FITC, and the mixture was gently vortexed. Then 10 μL of propidium iodide (PI) staining solution was added, mixed gently, and the cells were incubated at room temperature in the dark for 20 minutes. The cells were subsequently observed under a fluorescence microscope: Annexin V-FITC emits green fluorescence, while PI emits red fluorescence.

Bio-layer interferometry (BLI) assay

Binding kinetics of Rub and CPT1A were detected using BLI assay. In brief, the NTA sensor was

hydrated in buffer, and was bound to CPT1A human recombinant protein with His-tag. Then, the different concentrations of Rub solution were added to the sensor hole well to allow Rub to undergo binding reactions with the CPT1A human recombinant protein immobilized on the sensor. After the binding reaction, the sensor was transferred to the hole containing the buffer, so that the bound Rub was dissociated from the CPT1A human recombinant protein. Subsequently, according to the formula KD = kd /ka, the affinity constant KD value of Rub and CPT1A at different concentrations were calculated. Finally, the KD value was fitted to the Rub concentration to obtain the final affinity constant KD value through nonlinear regression analysis, which reflects the affinity between Rub and CPT1A.

Cellular thermal shift assay (CETSA)

LX2 cells and JS1 cells were cultured in 10 cm culture dishes and incubated with 150 μM Rub or DMSO for 2h. After incubation, the cells were washed with PBS, lysed by RIPA lysis buffer, centrifuged at 340 g for 5 min, and the supernatant was collected. Subsequently, the cell lysates were divided into several equal parts and incubated in a thermal cycler at a temperature range of 45 - 70°C, namely 45°C, 50°C, 55°C, 60°C, 65°C, 70°C. Each temperature point was set with at least 3 multiple wells and incubated for 15 min. Next, the samples were quickly placed on ice to cool for 10 min to terminate the heat-induced protein structure changes. The cooled samples were centrifuged to take the supernatant, and the protein concentrations were quantified by BCA method, and finally the protein expression of CPT1A was detected by western blot assay.

Drug affinity responsive target stability (DARTS) assay

LX2 and JS1 cells were lysed using RIPA lysis buffer with protease inhibitor, disrupted by ultrasound and centrifuged at 12, 000 rpm for 15 min. Centrifugal supernatants were incubated with DMSO or different concentrations of Rub (50, 100, 150 μM) at room temperature for 1 h, and then divided into several equal parts. The protease solution was added to the sample (protease: protein = 1:500) and the same volume of buffer as the protease was added to the control sample. Next, these samples were incubated at room temperature for 15 min, protease inhibitor was added to each sample and reacted at room temperature for 10 min. SDS-PAGE loading buffer were added to terminate reaction and prepared into the protein loaded samples. Finally, the protein expression of CPT1A was detected by

western blot assay.

Ubiquitination assay

To investigate the effect of Rub on CPT1A or c-Myc ubiquitination, activated LX2 or JS1 cells were treated with either Rub-PROTAC or Rub or Etomoxir for 10 or 48 h. Cells were then lysed using ice-buffer supplemented with protease inhibitors. The clarified lysates were immunoprecipitated with anti-CPT1A and c-Myc antibody for 1 h at room temperature, followed by overnight incubation with protein A/G magnetic beads at 4°C. After extensive washing with ice-cold PBS, immunoprecipitated proteins were eluted and analyzed by western blot assay to detect CPT1A and c-Myc ubiquitination.

Molecular dynamics simulation

Molecular dynamics simulations were carried out using GROMACS (version 2022.6, GPU-accelerated with single-precision FFTW). The CHARMM36 all-atom force field was applied to generate the CPT1A topology, while the CGenFF server (version 1.0) was utilized to derive parameters for Rub. The protein-ligand complex was solvated in a cubic water box (10 Å buffer) using the TIP3P explicit water model. System neutrality was achieved by adding counterions, followed by the inclusion of 0.15 M KCl to maintain physiological isotonicity. Energy minimization was performed using the steepest descent algorithm (20,000 steps) to relieve steric clashes. Subsequently, the system underwent equilibration in two phases: 10 ps NVT ensemble (constant particle number, volume, and temperature) at 300 K using the V-rescale thermostat, followed by 10 ps NPT ensemble (constant pressure, 1.013 bar) with the Parrinello-Rahman barostat. Production MD simulations were executed for 200 ns under periodic boundary conditions, with trajectory data saved every 200 ps (1000 frames total). Post-simulation analyses included removal of periodic artifacts, rotational/translational fitting, and calculation of root-mean-square deviation (RMSD) and binding free energy metrics. All visualizations and quantitative analyses were performed using GROMACS built-in tools and VMD (version 1.9.3). This integrated computational framework provides atomic-level insights into the conformational dynamics and thermodynamic stability of the CPT1A-Rub complex, elucidating potential mechanisms of molecular recognition and binding.

Molecular docking

A computational investigation was conducted using CPT1A as the target protein to evaluate the binding interactions of Rub through molecular docking and molecular dynamics (MD) simulations. The molecular docking experiments were performed using the AutoDock 4.2 software suite (http://autodock.scripps.edu/). The NMR structure of CPT1A (PDB ID: 2le3) was retrieved from the Protein Data Bank (https://www.rcsb.org/). The ligand structure of Rub (PubChem CID: 124219) was downloaded in .sdf format and converted to .pdb format using Chem3D 15.0. Prior to docking, all ligands were energy-minimized using Avogadro software (version 1.2.0) to optimize their geometries. Receptor and ligand preprocessing, including hydrogen atom addition and Gasteiger charge assignment, was performed using MGL Tools and AutoDock utilities to generate .pdbqt files. Water molecules, co-crystallized ligands, and heteroatoms were removed from the protein structure. The docking grid box was defined with dimensions of 55 × 55 × 55 Å^3 (x, y, z axes) to encompass the putative binding site. Grid parameter files (.gpf) and docking parameter files (.dpf) were generated via the AutoDock graphical interface. Flexible ligand docking was implemented using the Lamarckian Genetic Algorithm (LGA) with default parameters in AutoDock 4.2. Autogrid and AutoDock modules were employed to identify optimal binding conformations, with the lowest Gibbs free energy (ΔG, kcal/mol) complex selected for subsequent analysis. Receptor-ligand interaction patterns were visualized in 2D and 3D using PyMol (version 2.5.2).

Untargeted lipidomics assay

LX2 cells were collected with 50 μL of PBS, and added with 200 μL of a mixed solution of chloroform and methanol (2:1). The mixture was vortexed and centrifuged at 13,000 rpm for 10 min, and then 200 uL of the lower organic phase was transferred to a centrifuge tube. 50 uL of ultrapure water was added for extraction of the organic phase. After centrifugation, 100 uL of the lower organic phase was transferred to the centrifuge tube and dried with nitrogen gas. Subsequently, the sample was dissolved in a mixture of dichloromethane and methanol (1:1). Finally, the lipidomics of the samples was detected by LC-MS/MS (AB SCIEX QTRAP5500).

Synthesis and characterization of Rub-PROTAC

NMR spectra were acquired on a Bruker AVANCE III HD nuclear magnetic resonance

spectrometer, running at 400 MHz and 600 MHz for 1H, 101 MHz and 151 MHz for 13C. 1H NMR spectra were recorded in CHCl3-d, using residual CHCl3 (7.26 ppm) as the internal reference. 13C NMR spectra were recorded in CHCl3-d, using residual CHCl3 (77.16 ppm) as the internal reference. Mass spectrometry was performed using a Thermo Scientific QExactive mass spectrometer (ESI). Analytical grade solvents and commercially available reagents were used without further purification.

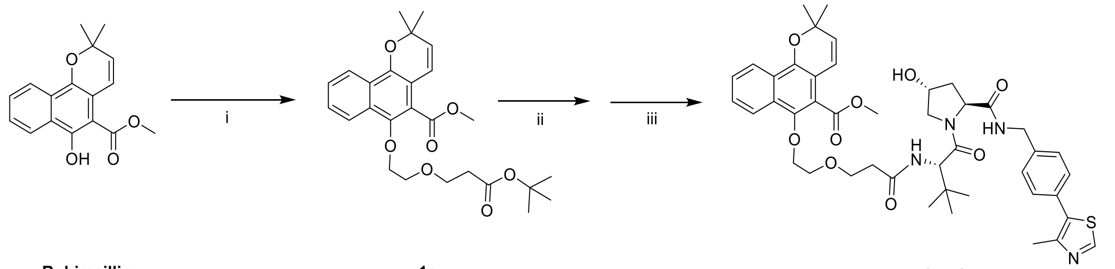

Scheme S1: Synthesis of Rub-PROTAC. Reagents and conditions: **(i)** *tert*-butyl 3-(2-bromoethoxy)propanoate, K_2CO_3, DMF, 80 °C, 1 h; **(ii)** HCl in 1,4-dioxane, R.T., 2 h; **(iii)** (S,R,S)-AHPC, HATU, DIPEA, DMF, R.T., 3 h. **(1a)** Methyl 6-(2-(3-(*tert*-butoxy)-3-oxopropoxy)ethoxy)-2,2-dimethyl-2H-benzo[h]chromene-5-carboxylate. **(Rub-PROTAC**) 6-(2-(3-(((S)-1-((2S,4R)-4-hydroxy-2-((4-(4-methylthiazol-5-yl)benzyl)carbamoyl)pyrrolidin-1-yl)-3,3-dimethyl-1-oxobutan-2-yl)amino)-3-oxopropoxy)ethoxy)-2,2-dimethyl-2H-benzo[h]chromene-5-carboxylate**.**

Synthesis of Rub-PROTAC was carried out in two steps (**Scheme S1)**, as follows: (1) Synthesis of methyl 6-(2-(3-(*tert*-butoxy)-3-oxopropoxy)ethoxy)-2,2-dimethyl-2H-benzo[h]chromene-5-carboxylate **(1a)**: A round - bottom flask was charged with Rubimaillin (300 mg, 1.0 equiv) and potassium carbonate (K_2CO_3, 218.7 mg, 1.5 equiv) under an atmosphere of nitrogen. Anhydrous N,N-dimethylformamide (DMF) was added as the solvent, followed by the addition of *tert*-butyl 3-(2 - bromoethoxy)propanoate (270.26 mg, 1.0 equiv). The reaction mixture was heated to 80°C and stirred for 1 h. After stirring for 1 h, the reaction mixture was allowed to reach room temperature. Then an appropriate amount of water and ethyl acetate were added for extraction. The combined organic layers were washed with brine and dried over anhydrous sodium sulfate, filtered, and concentrated under vacuum to give the crude product. The residue was purified on silica gel via petroleum ether : ethyl acetate = 5 : 1 to give a pale-yellow oily liquid **1a** (Methyl 6-(2-(3-(tert-butoxy)-3-oxopropoxy)ethoxy)-2,2-dimethyl-2H-benzo[h]chromene-5-carboxylate,270 mg, 56.1% yield). ^{1}H NMR (600 MHz, Chloroform-d) δ 8.19 – 8.12 (m, 2H),

7.48 (dd, J = 6.4, 3.3 Hz, 2H), 6.40 (d, J = 9.9 Hz, 1H), 5.66 (d, J = 9.9 Hz, 1H), 4.17 (t, 2H), 3.95 (s, 3H), 3.82 – 3.79 (m, 4H), 2.57 (t, J = 6.5 Hz, 2H), 1.49 (s, 6H), 1.45 (s, 9H). ^{13}C NMR (151 MHz, Chloroform-d) δ 170.97, 167.84, 146.34, 145.04, 130.22, 128.14, 126.91, 126.86, 126.77, 123.03, 122.35, 120.81, 119.91, 112.38, 80.65, 76.51, 75.14, 70.28, 67.18, 52.43, 36.49, 28.20, 27.78. HRMS (ESI) calculated $C_{26}H_{32}O_7$, $[M+H]^+$ = 456.2148, and measured $[M+H]^+$: 456.2139.

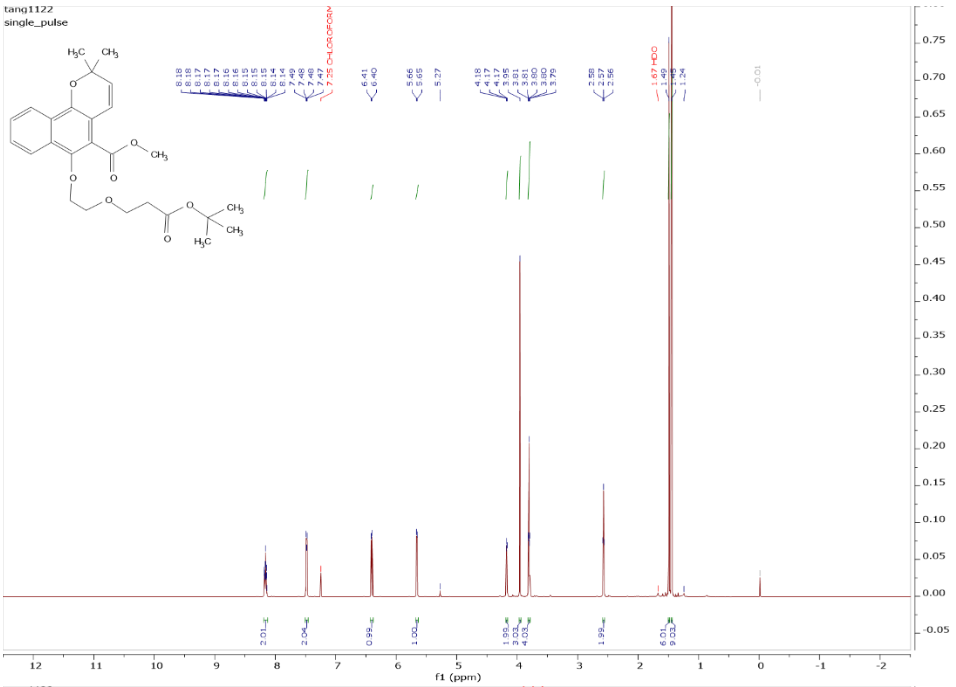

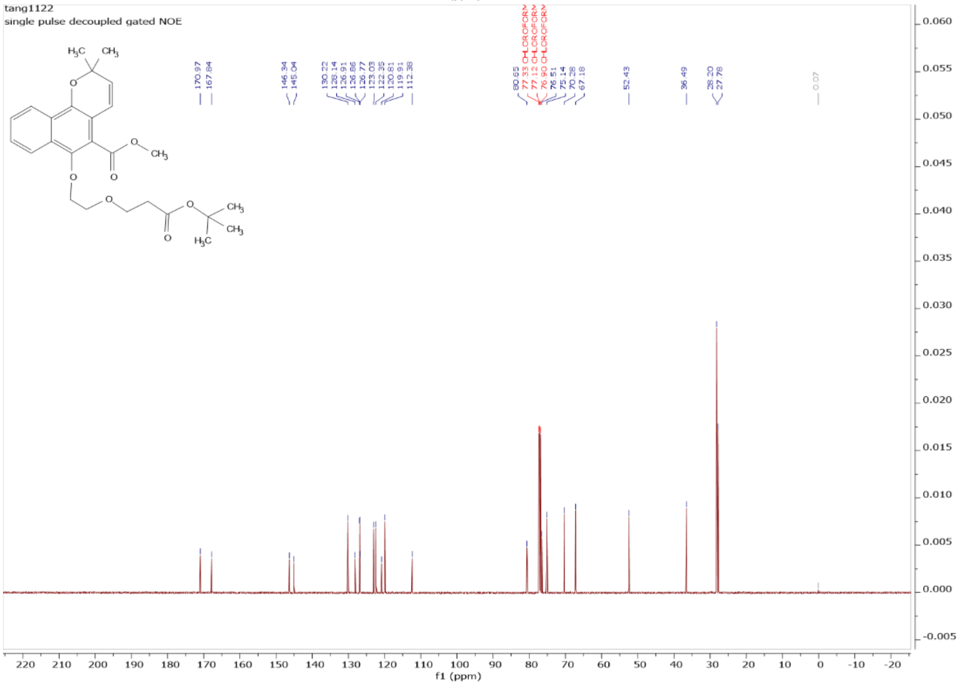

(2) Synthesis of methyl 6-(2-(3-(((S)-1-((2S,4R)-4-hydroxy-2-((4-(4-methylthiazol-5-

yl)benzyl)carbamoyl)pyrrolidin-1-yl)-3,3-dimethyl-1-oxobutan-2-yl)amino)-3-oxopropoxy)ethoxy)-2,2-dimethyl-2H-benzo[h]chromene-5-carboxylate **(Rub-PROTAC)**: A round-bottom flask was charged with compound Methyl 6-(2-(3-(*tert*-butoxy)-3-oxopropoxy)ethoxy)-2,2-dimethyl-2H-benzo[h]chromene-5-carboxylate **1a** (270 mg, 1.0 equiv). Then 5 mL of 1 M hydrochloric acid in 1,4 - dioxane solution was added drop-wise over 10 minutes. The mixture was continuously stirred until a white suspension was formed. The solvent was evaporated under reduced pressure, and the residue was dried under vacuum to give the crude product as a yellow solid, which was used in the next step without further purification. Anhydrous N,N-dimethylformamide (DMF) was added as the solvent, under continuous stirring at room temperature, (S,R,S)-AHPC (280 mg, 1.1 equiv), 1-[bis(dimethylamino)methylene]-1H-1,2,3-triazolo[4,5-b]pyridinium 3-oxide hexafluorophosphate (HATU, 270 mg, 1.2 equiv), and N,N-diisopropylethylamine (DIPEA, 230 mg, 3 equiv) were sequentially added to the above solution over 3 minutes. After the addition was completed, the mixture was stirred at room temperature for another 3 hours. Then an appropriate amount of water and ethyl acetate were added for extraction. The combined organic layers were washed with brine and dried over anhydrous sodium sulfate, filtered, and concentrated under vacuum to give the crude product. The residue was purified on silica gel via petroleum ether : ethyl acetate = 1 : 1 to give a pale-yellow solid **Rub-PROTAC** (350 mg, 72.7% yield). ^{1}H NMR (400 MHz, Chloroform-*d*) δ 8.69 (s, 1H), 8.24 – 8.16 (m, 1H), 8.14 – 8.05 (m, 1H), 7.53 – 7.45 (m, 2H), 7.40 (t, *J* = 6.0 Hz, 1H), 7.35 – 7.24 (m, 4H), 7.09 (d, *J* = 8.1 Hz, 1H), 6.41 (d, *J* = 9.9 Hz, 1H), 5.69 (d, *J* = 9.9 Hz, 1H), 4.71 (t, *J* = 7.9 Hz, 1H), 4.58 – 4.44 (m, 3H), 4.29 (d, *J* = 5.2 Hz, 1H), 4.24 (q, *J* = 4.6 Hz, 2H), 4.07 (d, 1H), 3.96 (s, 3H), 3.88 – 3.81 (m, 4H), 3.58 (dd, *J* = 11.3, 3.7 Hz, 1H), 2.61 – 2.46 (m, 6H), 2.09 (d, 1H), 2.06 (s, 3H), 1.51 (s, 6H), 0.90 (s, 9H). ^{13}C NMR (101 MHz, Chloroform-d) δ 172.09, 171.83, 171.12, 170.71, 167.77, 146.17, 145.04, 138.14, 130.29, 129.44, 128.07, 126.89, 126.84, 126.69, 122.78, 122.41, 120.71, 119.73, 76.51, 74.68, 70.43, 70.12, 67.27, 58.29, 57.85, 56.54, 52.40, 43.16, 36.75, 35.75, 34.70, 27.71, 26.35, 14.19. HRMS (ESI) calculated $C_{44}H_{53}N_4O_9S$, $[M+H]^+$ = 813.3533, and measured $[M+H]^+$: 813.3546.

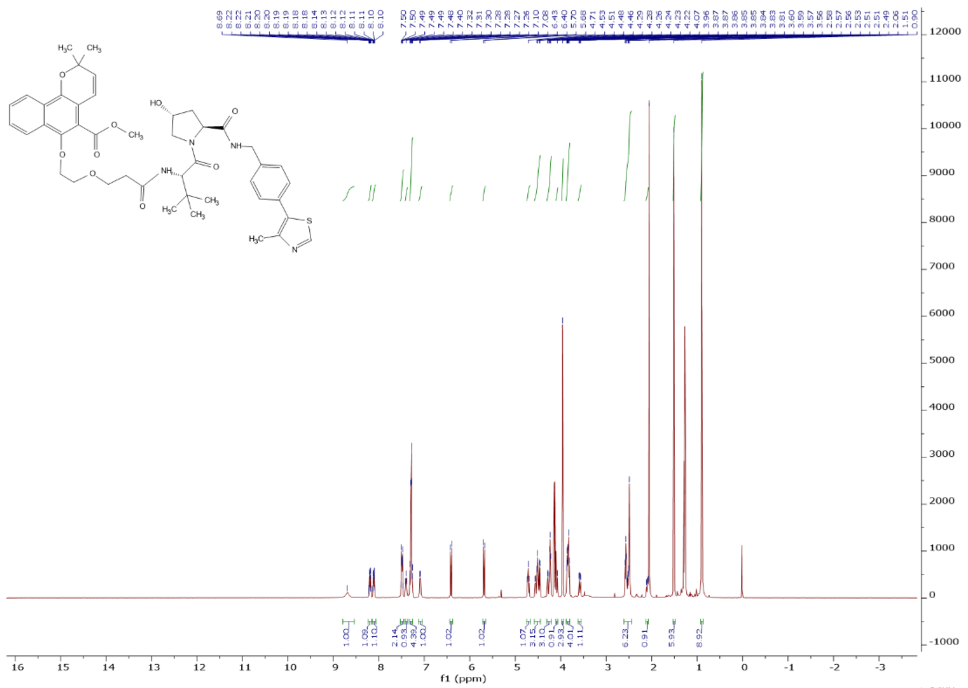

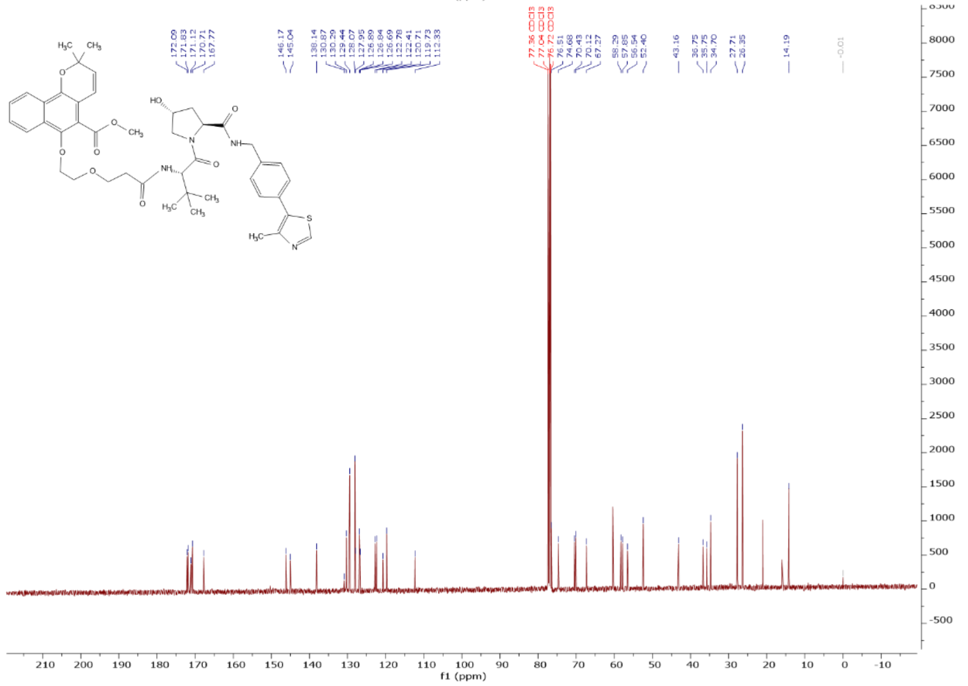

Table. S1 Primer sequences

Gene	Forward primers (5'-3')	Reverse primers (5'-3')
Human GAPDH	GTCTCCTCTGACTTCAACAGCG	ACCACCCTGTTGCTGTAGCCAA
Human α-SMA	CTATGCCTCTGGACGCACAACT	CAGATCCAGACGCATGATGGCA
Human Col1A1	GATTCCCTGGACCTAAAGGTGC	AGCCTCTCCATCTTTGCCAGCA
Mouse GAPDH	CATCACTGCCACCCAGAAGACTG	ATGCCAGTGAGCTTCCCGTTCAG
Mouse α-SMA	TGCTGACAGAGGCACCACTGAA	CAGTTGTACGTCCAGAGGCATAG
Mouse Col1A1	CCTCAGGGTATTGCTGGACAAC	CAGAAGGACCTTGTTTGCCAGG
Mouse Col3A1	GACCAAAAGGTGATGCTGGACAG	CAAGACCTCGTGCTCCAGTTAG
Mouse TGF-β1	TGATACGCCTGAGTGGCTGTCT	CACAAGAGCAGTGAGCGCTGAA

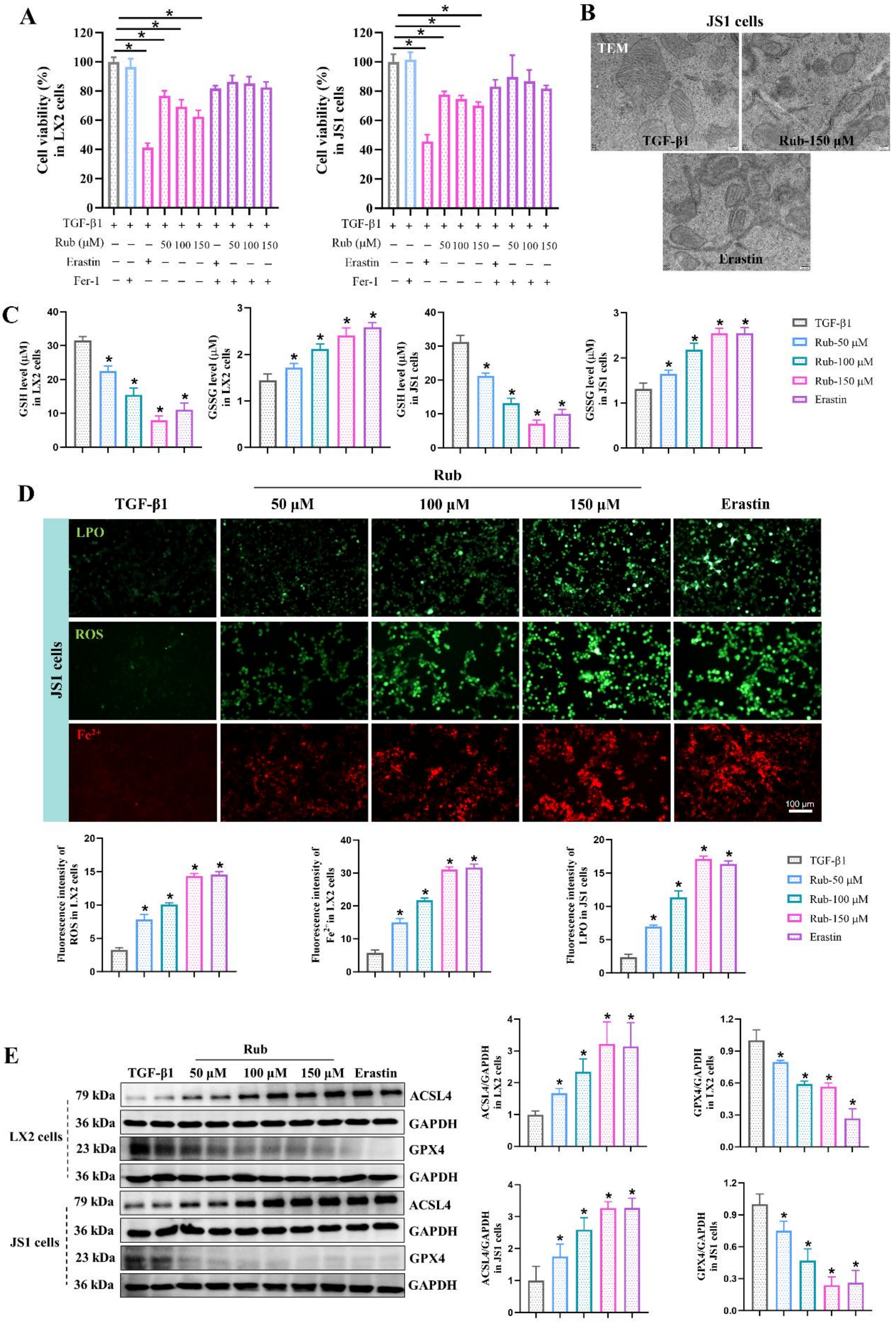

Figure S1. The effect of Rub on the ferroptosis of activated HSC *in vitro*. (A) Cell viability of Rub, Erastin and Fer-1 in LX2 and JS1 cells with TGF-β1. (B) the changes of mitochondrial ultrastructure in

TGF-β1-induced JS1 cells (scale bar=200 nm). (C) The levels of GSH and GSSG in TGF-β1-induced LX2 and JS1 cells. (D) Fluorescence staining of LPO, ROS, and Fe^{2+} in TGF-β1-induced JS1 cells (scale bar = 100 μm). (E) The protein expressions of ACSL4 and GPX4 in TGF-β1-induced LX2 and JS1 cells. All experiments were performed with n = 3 independent biological replicates. $^{\#}p < 0.05$ *vs* Control ; $^{*}p < 0.05$ *vs* TGF-β1.

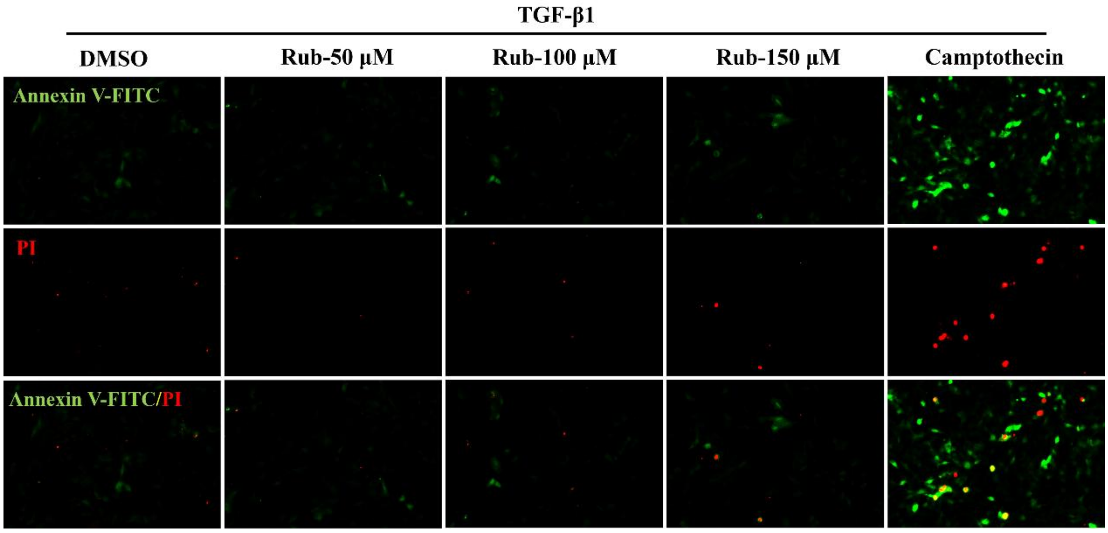

Figure S2. The effect of Rub on the apoptosis or necrosis of activated HSC *in vitro*. Annexin V-FITC^{+}/PI^{-}: apoptotic cells, Annexin V-FITC^{+}/PI^{+}: necrotic cells. All experiments were performed with n = 3 independent biological replicates.

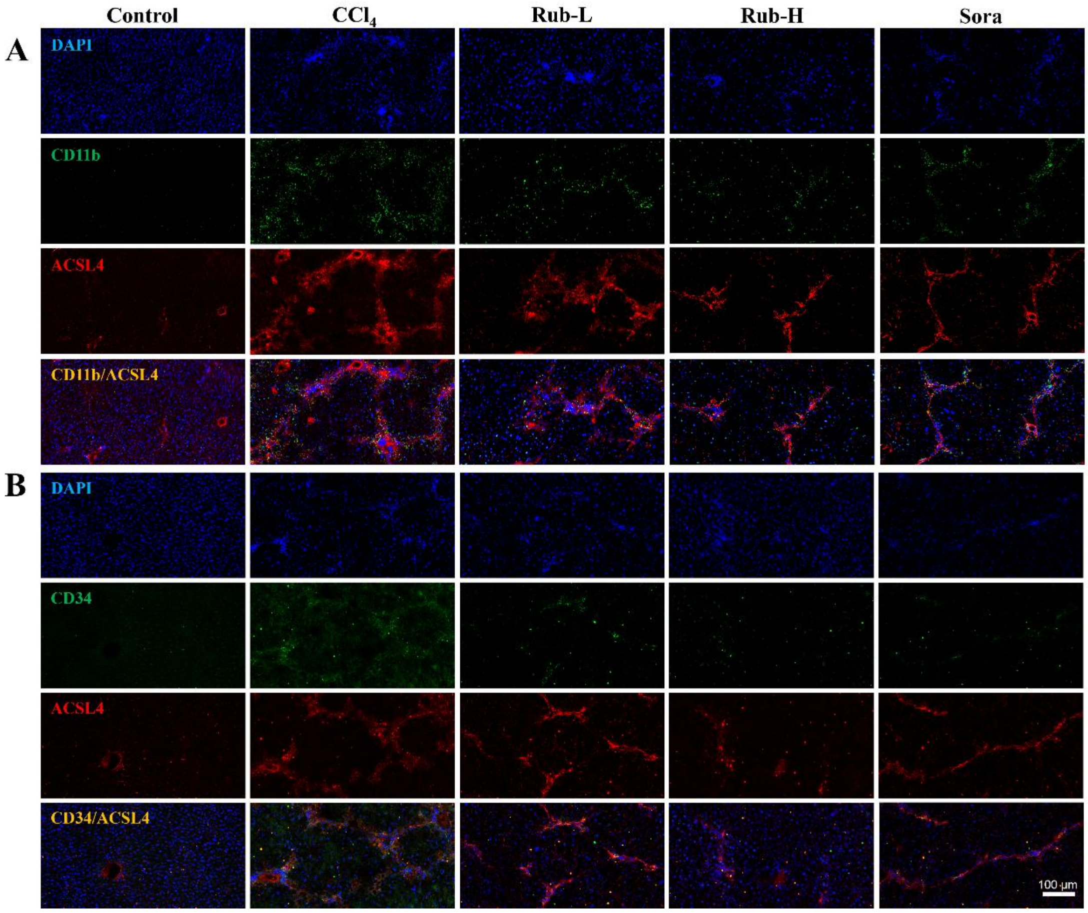

Figure S3. The effect of Rub on the ferroptosis of Kupffer and HSEC *in vivo*. (A) Immunofluorescence co-staining of ACSL4 (red) and CD11b (green) in CCl_4-induced mice liver sections (n = 3, scale bar = 100 μm). (B) Immunofluorescence co-staining of ACSL4 (red) and CD34 (green) in CCl_4-induced mice liver sections (n = 3, scale bar = 100 μm).

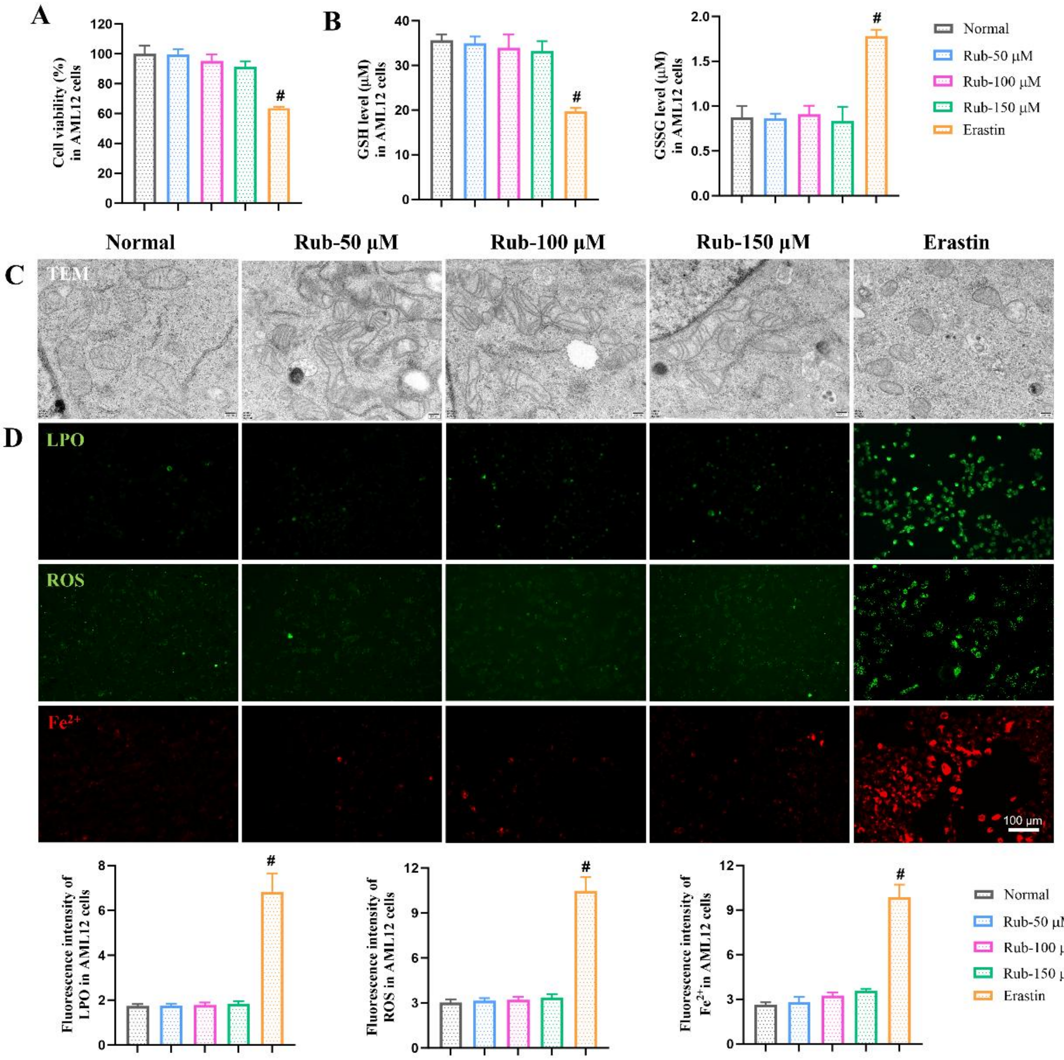

Figure S4. The effect of Rub on the ferroptosis in AML12 cells. (A) Cell viability of AML12 cells treated with different concentrations of Rub, with Erastin used as a positive control. (B) The levels of GSH and GSSG in AML12 cells. (C) The changes of mitochondrial ultrastructure in AML12 cells (scale bar = 200 nm). (D) Fluorescence staining of LPO, ROS, and Fe^{2+} in AML12 cells (scale bar = 100 μm). All experiments were performed with n = 3 independent biological replicates. $^{\#}p < 0.05$ *vs* Normal.

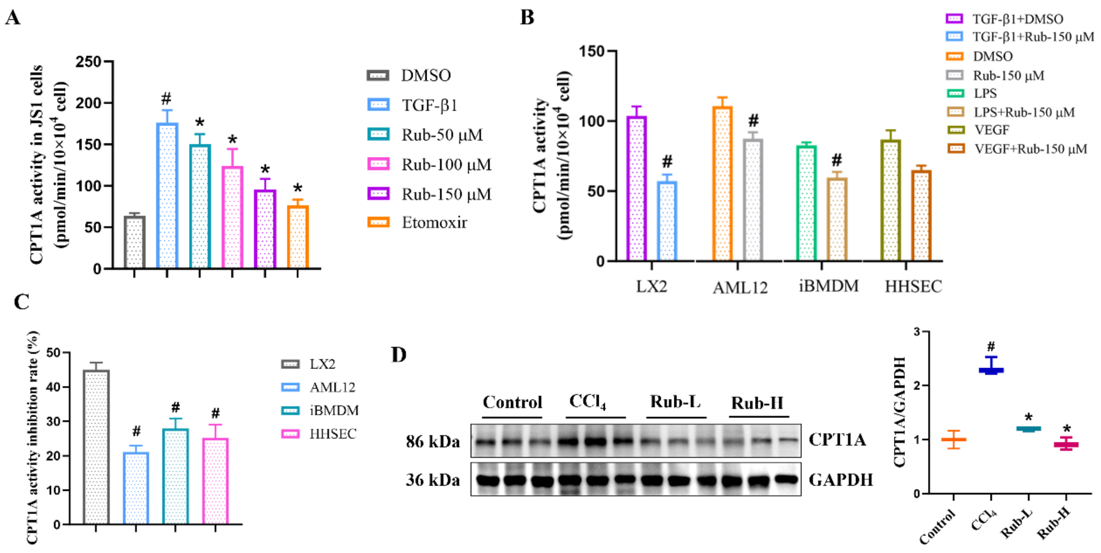

Figure S5. The effect of Rub on the activity and protein of CPT1A *in vitro* and *in vivo*. (A) The activity of CPT1A in JS1 cells. (C) The activity of CPT1A in LX2, AML12, iBMDM and HHSEC cells. (C) The activity inhibition rate of CPT1A in LX2, AML12, iBMDM and HHSEC cells. (D) The protein of CPT1A in mice. All experiments were performed with n = 3 independent biological replicates. $^{\#}p < 0.05$ *vs* DMSO or TGFβ1+DMSO or LPS or LX2 or Control, $^{*}p < 0.05$ *vs* CCl_4.

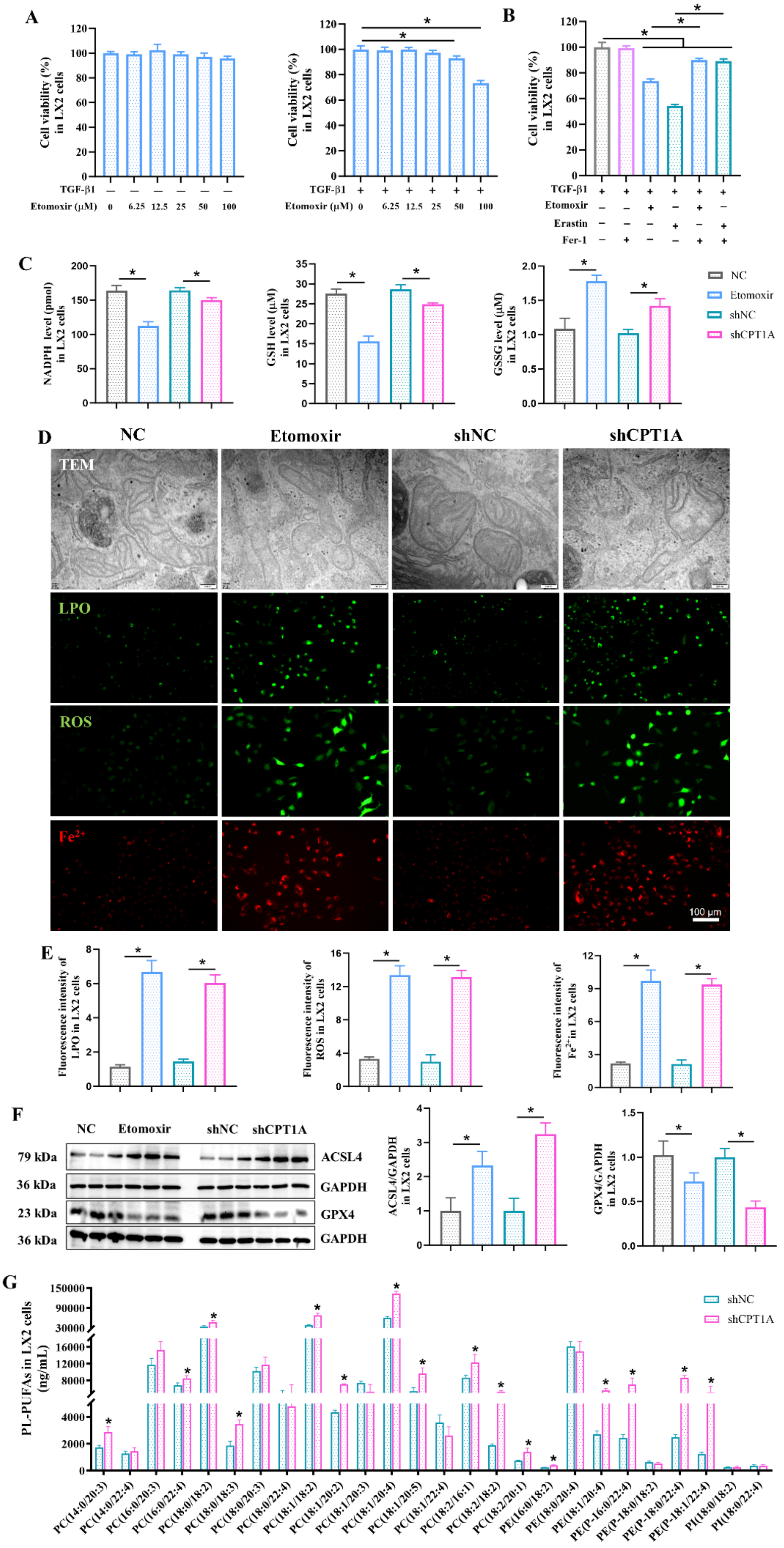
A
Cell viability (%) in LX2 cells
TGF-β1
Etomoxir (μM) 0 6.25 12.5 25 50 100
B
Etomoxir
Erastin
Fer-1
C
NADPH level (pmol) in LX2 cells
GSH level (μM) in LX2 cells
GSSG level (μM) in LX2 cells
NC
Etomoxir
shNC
shCPT1A
D
NC
Etomoxir
shNC
shCPT1A
TEM
LPO
ROS
Fe2+
100 μm
E
Fluorescence intensity of LPO in LX2 cells
Fluorescence intensity of ROS in LX2 cells
Fluorescence intensity of Fe2+ in LX2 cells
F
NC
Etomoxir
shNC
shCPT1A
79 kDa
ACSL4
36 kDa
GAPDH
23 kDa
GPX4
36 kDa
GAPDH
ACSL4/GAPDH in LX2 cells
GPX4/GAPDH in LX2 cells
G
PL-PUFAs in LX2 cells (ng/mL)
shNC
shCPT1A
PC(14:0/20:3)
PC(14:0/22:4)
PC(16:0/20:3)
PC(16:0/22:4)
PC(18:0/18:2)
PC(18:0/18:3)
PC(18:0/20:3)
PC(18:0/22:4)
PC(18:1/18:2)
PC(18:1/20:2)
PC(18:1/20:3)
PC(18:1/20:4)
PC(18:1/20:5)
PC(18:1/22:4)
PC(18:2/16:1)
PC(18:2/18:2)
PC(18:2/20:1)
PE(16:0/18:2)
PE(18:0/20:4)
PE(18:1/20:4)
PE(P-16:0/22:4)
PE(P-18:0/18:2)
PE(P-18:0/22:4)
PE(P-18:1/22:4)
PI(18:0/18:2)
PI(18:0/22:4)

Figure S6. The effect of CPT1A inhibition or deficiency on the ferroptosis of activated HSCs in LX2 cells. (A) Cell viability of Etomoxir in LX2 cells without or with TGF-β1. (B) Cell viability of Etomoxir, Erastin, and Fer-1 in LX2 cells with TGF-β1. (C) The levels of NADPH, GSH and GSSG in LX2 cells. (D) The changes of mitochondrial ultrastructure (scale bar = 200 nm), fluorescence staining of LPO, ROS, and Fe^{2+} (scale bar = 100 μm) in LX2 cells. (E) Fluorescence intensity analysis of LPO, ROS, and Fe^{2+} in LX2 cells. (F) The protein expressions of ACSL4 and GPX4 in LX2 cells. (G) The level changes of PL-PUFAs in LX2 cells. All experiments were performed with n = 3 independent biological replicates. $^{*}p < 0.05$ *vs* NC or shNC.

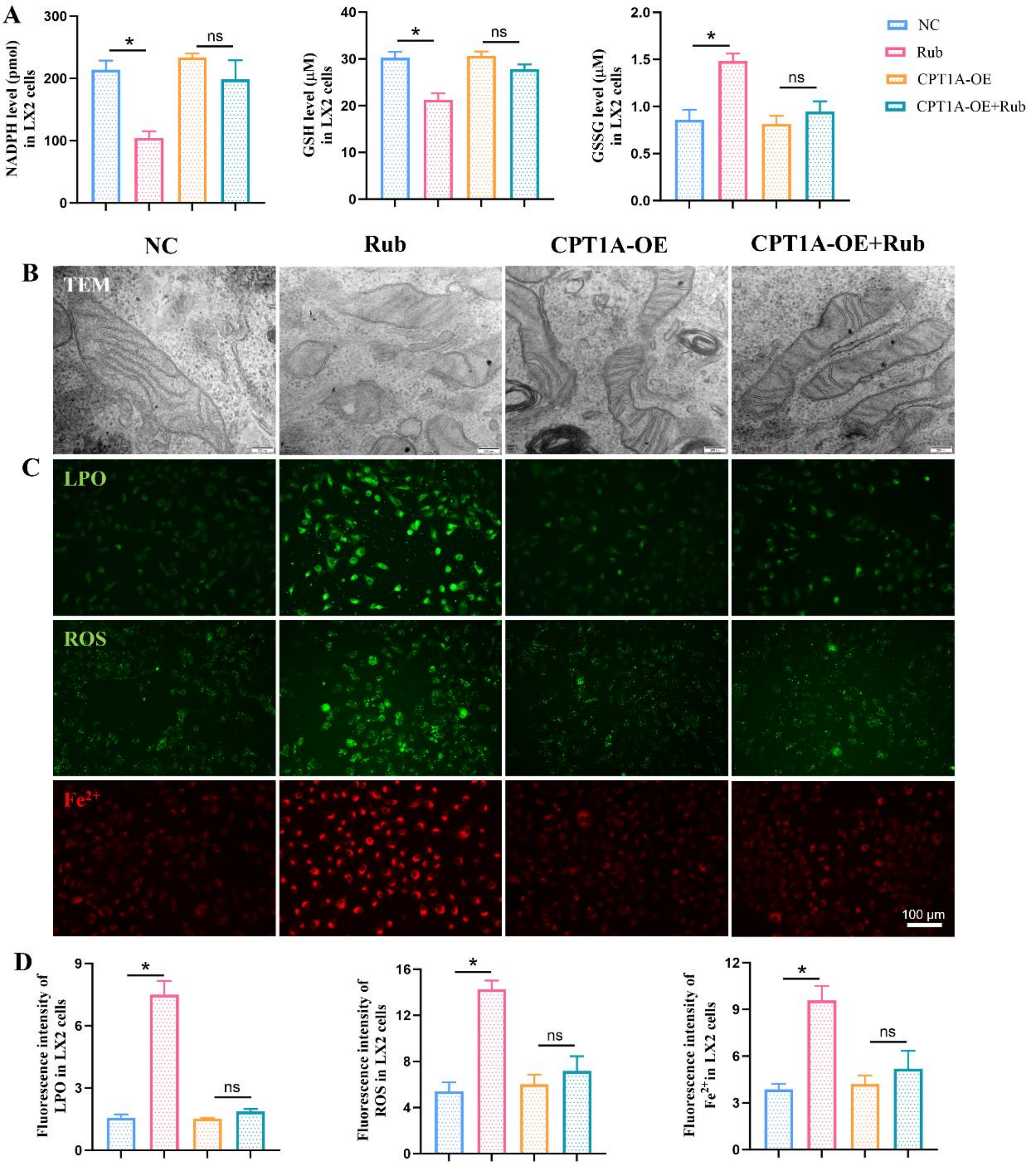

Figure S7. The effect of CPT1A overexpression on Rub-induced the ferroptosis in TGF-β1-induced LX2 cells. (A) The levels of NADPH, GSH and GSSG in LX2 cells. (B) The changes of mitochondrial ultrastructure in LX2 cells (scale bar = 200 nm). (C and D) Fluorescence staining of LPO, ROS, and Fe^{2+} in LX2 cells (scale bar = 100 μm). All experiments were performed with n = 3 independent biological replicates. $^{*}p < 0.05$ *vs* NC.
